# Cognitive Motivation as a Resource for Affective Adjustment and Mental Health

**DOI:** 10.3389/fpsyg.2021.581681

**Published:** 2021-09-21

**Authors:** Alexander Strobel, Aniko Farkas, Jürgen Hoyer, Ursula Melicherova, Volker Köllner, Anja Strobel

**Affiliations:** ^1^Differential and Personality Psychology, Faculty of Psychology, Technische Universität Dresden, Dresden, Germany; ^2^Division of Personality Psychology and Assessment, Department of Psychology, Chemnitz University of Technology, Chemnitz, Germany; ^3^Behavioural Psychotherapy, Faculty of Psychology, Technische Universität Dresden, Dresden, Germany; ^4^Psychosomatic Rehabilitation Research Group, Department of Psychosomatic Medicine, Charité – Universitätsmedizin Berlin, Berlin, Germany; ^5^Department of Psychosomatic Medicine, Rehabilitation Center Seehof, Federal German Pension Agency, Teltow, Germany

**Keywords:** Need for Cognition, depressive symptoms, effortful control, self-regulation, inhibition, attention, emotion regulation, cognitive motivation

## Abstract

**Background:** Depressive symptoms compromise cognitive and self-regulating capacities. Overcoming associated deficits (e.g., attentional bias) demands cognitive effort and motivation. Previous studies on healthy individuals have found cognitive motivation to positively relate to self-regulation and negatively to depressive symptoms. A test of these associations in a clinical sample is lacking.

**Methods:** We assessed cognitive motivation, self-regulation and depressive symptoms by means of well-validated questionnaires in *N* = 1,060 psychosomatic rehabilitation in-patients before and after treatment. Data were split and analyzed in two steps: We tested previously reported cross-sectional and longitudinal associations of all variables as well as their longitudinal changes in a first sample. Afterward, findings and derived hypotheses were replicated and tested in a second sample.

**Results:** Analyses of both samples confirmed earlier reports on positive associations between cognitive motivation and self-regulation, and negative associations of both with depressive symptoms. While the change in all variables was predicted by their baseline scores, higher baseline cognitive motivation was found to predict stronger improvements in self-regulation, and lower baseline depression scores to predict smaller changes in cognitive motivation and self-regulation. In addition, the change in cognitive motivation partially mediated the association between the changes in depressive symptoms and self-regulation.

**Conclusion:** Based on a large longitudinal data set, the present study expands previous findings and suggests a resource allocation model in which decreasing depressive symptoms lead to a release of capacities benefitting self-regulation directly, and indirectly *via* cognitive motivation.

## Introduction

Depression is one of the most common and impairing mental disorders of the modern world (World Health Organization, 2017). Individuals suffering from this disorder have to cope with deficits in cognitive control and emotion regulation that manifest in depressive rumination and cognitive, particularly attentional bias (Gotlib and Joormann, 2010; Joormann and Gotlib, 2010; Disner et al., 2011). Empirically supported interventions such as cognitive-behavioral therapy (CBT) and behavioral activation (BA) thus aim to identify and modify the maladaptive cognitive and behavioral patterns that might contribute to the development and maintenance of depressive symptoms (Mazzucchelli et al., 2009; Hofmann et al., 2012; Farmer and Chapman, 2016). The process of changing dysfunctional response patterns relies on a broad variety of factors including patient, therapist, and setting variables. This article focuses on the patient and its capacity to exert self- and effortful control.

### Personality Aspects Related to Adaptive Adjustment

*Self-control* is defined as “the ability of the self to alter its own responses, including thoughts, emotions, impulsive behavior, and performances” (Baumeister, 2002, p. 129). It is assumed to represent “one of the most distinctively human traits” (Baumeister et al., 2006, p. 1773) and to be an essential basis for adaptive behavior and affective adjustment (Tangney et al., 2004; Bertrams and Dickhäuser, 2012). Accordingly, it has been associated with better academic and social outcomes as well as reduced vulnerability for psychopathological syndromes (Tangney et al., 2004; Baumeister et al., 2006).

Being closely related to self-control, temperamental *effortful control* refers to the “efficacy of executive attention” (Rothbart and Bates, 2006, p. 129). By enabling individuals to “willfully or voluntarily inhibit, activate, or change (modulate) attention and behavior” (Eisenberg et al., 2011, p. 263), effortful control is fundamental for adaptive self- and emotion regulation (Rothbart and Rueda, 2005; Eisenberg et al., 2011). In this respect, deficits in attentional and particularly inhibitory control have been associated with depression-related difficulties in disengaging from negative stimuli and impaired emotion regulation typically manifesting in attentional (and memory) bias as well as rumination (Joormann and Gotlib, 2010; Disner et al., 2011; Koster et al., 2011). In order to overcome these dysfunctional responses, realignment of attention and engagement in adaptive behavior (e.g., reappraisal) would be needed (Joormann, 2010). This requires effortful cognitive control and thus sufficient cognitive capacity (Gotlib and Joormann, 2010; Joormann, 2010; Disner et al., 2011). Cognitive and particularly attentional capacity, however, is limited in both healthy and depressed individuals (Kahneman, 1973). Beyond that, emotional states have been assumed to influence the magnitude of allocable attentional capacity (Ellis and Ashbrook, 1988). In this respect, particularly the regulation of aversive emotional (depressive) states has been argued to reduce the availability of (attentional) resources and thus their allocation to processes that might also rely on them (Ellis and Ashbrook, 1988; Koole, 2009; Tice, 2009; Gotlib and Joormann, 2010; Krämer et al., 2014). This may hamper individuals with depression to engage in other effortful, but adaptive behavioral as well as cognitive processes that might help reducing symptoms (Gotlib and Joormann, 2010; Joormann, 2010). Thus, in order to enable more functional responses, (attentional) capacities would have to be sufficiently available and/or adequately (re-)allocated.

Besides emotional states, *motivation* has been found to affect the (re)alignment of attention as well (Engelmann and Pessoa, 2007; Pessoa, 2009; Nishiguchi et al., 2016). Corresponding studies have found motivation to influence and improve the efficiency of (re)orientating attention by determining the allocation of executive processing resources (Engelmann and Pessoa, 2007; Pessoa, 2009). Interestingly, allocating cognitive resources (or cognitive effort) has been considered the essence of interindividual differences in a particular form of motivation: Cognitive motivation (Enge et al., 2008). In this respect, *Need for Cognition (NFC)* –a core aspect of cognitive motivation (Cacioppo et al., 1996; von Stumm and Ackerman, 2013)– was found to be associated with voluntary as well as automatic attention allocation (Enge et al., 2008).

NFC refers to an individual’s tendency “to engage in and enjoy effortful cognitive endeavors” (Cacioppo et al., 1996, p. 197). Higher NFC has been associated with superior cognitive and academic performance (e.g., Cacioppo et al., 1996; von Stumm and Ackerman, 2013). Beyond that, NFC has been shown to be beneficial for affective adjustment and well-being (Bye and Pushkar, 2009; Bertrams and Dickhäuser, 2012; Strobel et al., 2017; Grass et al., 2018). The positive effects of NFC on affective well-being have been shown to be mediated by adaptive forms of self- and emotion regulation (Bye and Pushkar, 2009; Bertrams and Dickhäuser, 2012; Grass et al., 2018). Individuals with high NFC display higher self- (Bertrams and Dickhäuser, 2012) and effortful control (Nishiguchi et al., 2016), and apply adaptive coping (Bye and Pushkar, 2009) as well as emotion regulation strategies (e.g., reappraisal) (Strobel et al., 2017; Grass et al., 2018), which contribute to affective adjustment and positive emotionality.

### Associations Between Self-Regulation, Motivation and Depression

A few studies have investigated the association of NFC with self-regulation (self- or effortful control) and depressive symptoms in healthy individuals: The study by Bertrams and Dickhäuser (2012) found NFC to positively relate to self-control, and NFC and self-control to negatively relate to depressive mood. In addition, self-control was shown to completely mediate the relationship between NFC and affective adjustment (operationalized by self-esteem and habitual depressive mood). A study by Nishiguchi et al. (2016) investigated cross-sectional and longitudinal associations of NFC, effortful control and subclinical depression. Besides showing NFC to positively relate to effortful control, and NFC and effortful control to negatively relate to depressive symptoms, the authors found NFC to reciprocally interact with effortful control over time and to (partially) mediate and moderate the negative association between depressive symptoms and effortful control. On this basis, the authors concluded that NFC might mitigate the adverse effects depressive symptoms may have on attentional functions (Nishiguchi et al., 2016).

### Purpose of the Present Study

The findings of both studies are in line with the reports on reduced motivation and cognitive impairments in depression (e.g., Scheurich et al., 2008) and suggest potential ways by which depressive symptoms may interfere with effortful cognitive processes. The clinical relevance of these findings, however, is strongly limited as both studies were based on healthy undergraduates. Furthermore, analyses in these studies were mainly based on cross-sectional data. In order to identify the potential mechanisms that might contribute to the observed effects, longitudinal data would be preferable. Nishiguchi et al. (2016) provided some, but did not use them consistently in their analyses as their mediation model was based on cross-sectional data. Beyond that, although computing (residualized) change scores, the authors reported only selected correlations between baseline and change scores (those between baseline and corresponding change scores were missing). And even though these associations provided the basis for their longitudinal analyses, only effects of baseline scores on follow-up scores were investigated in their longitudinal model. This procedure, however, does not allow to comprehensively examine whether symptoms and traits had significantly changed from the first to the second assessment, and if so, how these changes might be related to each other as well as to the baseline characteristics. Nevertheless, Nishiguchi et al. (2016) put forward a model, according to which cognitive motivation (NFC) mediated the negative impact of depressive symptoms on self-regulation (effortful control). As underlying mechanism, the authors pointed to the effects of cognitive motivation on attentional processes (Nishiguchi et al., 2016). Following this line of argumentation, it was assumed here that, presumably in order to (re-)gain an affective equilibrium, depressive states temporarily compromise self-regulating and cognitive capacities by occupying or limiting the amount of available attentional resources (Ellis and Ashbrook, 1988). By selectively attending to negative stimuli and trying to “ruminate” their way out, these resources might rather be dysfunctionally deployed though and thereby contribute to the development and perpetuation of symptoms (Joormann and Gotlib, 2010).

Thus, in order to encounter and overcome such maladaptive patterns, resources that are bound by depression have to be restored to enable individuals to deliberately modulate their attentional, emotional and behavioral responses in an adaptive way. This is why strengthening effortful self-control (and motivation), particularly the realignment of resources toward adaptive processes, is such an essential goal and requirement in the treatment of depression.

With decreasing depressive symptoms, so might do as well the corresponding executive (attentional) resource demands. Capacities that have so far been employed rather maladaptively, might subsequently become available for initiating more adaptive effortful processes. This resource shift might lead to the re-allocation of the capacities pertaining to self-regulation and cognitive motivation toward a more functional realignment of executive attention and thereby contribute to improved self-regulation.

In order to examine the presumed shifts in resources that might underlie depression-related impairments as well as treatment-related improvements in effortful cognitive and self-regulating processes, it would be necessary to investigate the longitudinal changes in all variables as well as their interdependencies among each other and with the baseline characteristics. It was thus the aim of the present study to investigate cognitive motivation, self-regulation and depressive symptoms comprehensively in a clinical sample before and after treatment.

For this purpose, we applied a two-step procedure: On the basis of the findings delineated above, we firstly intended to investigate the cross-sectional associations of cognitive motivation, self-regulation, and depressive symptoms. With respect to the longitudinal associations, we also referred to previous studies, but pursued a different analyzing approach by establishing a model using latent change score modeling instead of path analysis. This allowed us to examine if and in what way variable characteristics and corresponding resource allocation would change over the course of treatment. For sample 1, we thus cross-sectionally assumed that cognitive motivation and self-regulation would be positively intercorrelated and negatively related to depressive symptomatology. Longitudinally, we hypothesized that baseline scores of all variables would predict their corresponding change scores. We further assumed that baseline cognitive motivation would predict changes in self-regulation, and baseline self-regulation to predict changes in cognitive motivation as well as depressive symptoms at the second assessment. The findings of sample 1 provided the ground for revising and differentiating our initial hypotheses and established models, which were then tested in the second step in the second sample.

## Sample 1: Derivation of the Model

### Materials and Methods

We report how we determined our sample size, all data exclusions, all manipulations, and all measures in the study (cf. Simmons et al., 2012). All data, analyzing scripts and materials for reproducing our analyses are permanently and openly accessible at https://osf.io/e8tg5 (Strobel et al., 2020). The analyses of the first sample were not pre-registered.

#### Participants and Procedure

This study was conducted in preparation of a multi-center clinical trial, which was approved by the Federal German Pension Agency (#8011-106-31/31.127). Participants were recruited from in-patients of a psychosomatic rehabilitation center. Adults who underwent inpatient psychosomatic rehabilitation between February 2018 and April 2019 were informed about the study during their clinic admission. Participating patients had to be suitable for rehabilitation treatment, which required them to not being suicidal or psychotic, be able to attend therapy (particularly group) sessions, and to have a good prognosis of regaining work ability (Köllner, 2016). As this study intended to explore the association of cognitive motivation and self-regulation with depressive symptoms, only individuals scoring above 8 on the BDI-II scale were included in the final analyses (Hautzinger et al., 2009).^1^ Several of the patients received antidepressants in accordance with the guidelines for rehabilitation treatment, but did not receive any psychotropic medication that could alter cognitive functioning. Patients who agreed to participate gave their written consent. The study was approved by the local ethics committee of Chemnitz University of Technology (V-250-15-AS-MOTIVATION-15012018). Data were collected under the supervision of a psychological technical assistant during the computer-based routine diagnostic procedure that each patient mandatorily completed before (t_1_) and after (t_2_) treatment.

The total sample consisted of 1,060 patients (66.89% females) with a mean age of 51.69 years (*SD* = 8.67). For analyzing purposes (see section “Statistical Analysis”), the total sample was split into two samples of 530 individuals each. At first, only the data from the first sample were analyzed, of which 35 individuals had to be excluded due to missing values and further 36 cases due to subthreshold BDI-II scores (see section “Statistical Analysis”). Mean age of this sample of *n*_1_ = 459 individuals (67.54% females) was 52.05 years (*SD* = 8.43). Due to varying individual durations of stay and corresponding times of discharge, first and second assessment were conducted within a median time distance of 33 days (range: 14–77 days).

#### Measures

##### Beck Depression Inventory, Revised (BDI–II; Hautzinger et al., 2009)

Depressive symptoms were assessed with the German version of the revised Beck Depression Inventory (BDI–II; Hautzinger et al., 2009). By means of 21 items the BDI–II assesses presence and severity of the main diagnostic criteria of depressive disorders (e.g., depressive mood, loss of interest) according to DSM–IV (American Psychiatric Association, 1994; Hautzinger et al., 2009). Participants are asked to retrospectively rate their symptoms within the last 14 days by choosing one of four answer options (Hautzinger et al., 2009), each of which is rated from zero to three. Numbers of all answers are aggregated into a total sum score, which indicates the severity of the symptoms from not existing (0–8) over minimal (9–13), light (14–19) and moderate (20–28) to severe (29–63). The Cronbach’s alpha for sample 1 and sample 2 (t_1_: α = 0.89; t_2_: α = 0.94) are reported in Table 1 and corresponded to previous studies (Hautzinger et al., 2009; Nishiguchi et al., 2016).

**TABLE 1:** Descriptive statistics and correlation coefficients between t_1_- and t_2_-scores of all measures.

	COM_t_1_	COM_t_2_	ESC_t_1_	ESC_t_2_	CEI_t_1_	CEI_t_2_	BDI_t_1_	BDI_t_2_	*Mean*	*SD*	Range
COM_t_1_	(0.90) (0.88)	**0.83**	0.53	0.50	0.91	0.77	−0.42	−0.32	1.90	15.21	−36–36
COM_t_2_	**0.80**	(0.76) (0.77)	0.45	0.59	0.76	0.92	−0.43	−0.46	3.01	14.85	−35–36
ESC_t_1_	0.54	0.41	(0.89) (0.89)	**0.73**	0.82	0.63	−0.51	−0.39	0.02	11.33	−34–29
ESC_t_2_	0.51	0.57	**0.72**	(0.90) (0.90)	0.67	0.84	−0.50	−0.54	2.27	11.09	−33–28
CEI_t_1_	0.91	0.71	0.84	0.68	(0.91) (0.91)	**0.81**	−0.52	−0.40	1.92	23.29	−54–53
CEI_t_2_	0.76	0.91	0.61	0.85	**0.79**	(0.78) (0.81)	−0.51	−0.55	5.28	23.24	−66–62
BDI_t_1_	−0.36	−0.33	−0.41	−0.41	−0.43	−0.41	(0.89) (0.89)	**0.68**	27.05	10.75	9–58
BDI_t_2_	−‘0.34	−0.43	−0.37	−0.55	−0.39	−0.54	**0.67**	(0.94) (0.94)	13.91	11.84	0–55
*Mean*	2.22	2.80	−0.24	2.03	1.98	4.83	26.90	13.36	–	–	–
*SD*	14.08	14.14	11.30	11.50	22.47	22.98	10.36	11.52	–	–	–
*Range*	−36–35	−36–36	−32–30	−33–36	−68–59	−69–72	9–56	0–50	–	–	–

*Analyses of the first (*n*_1_ = 459) and second (*n*_2_ = 468) sample.*

*Means, standard deviations, range of scores and Spearman’s Rank coefficients of the first sample are displayed above, of the second sample below the diagonal.*

*Internal consistencies (Cronbach’s alpha) are presented in the diagonal (upper line: first sample, lower line: second sample).*

*Bold-faced coefficients give the retest-reliabilities (interval on average 5 weeks).*

*BDI, depressive symptoms; COM, cognitive motivation; ESC, effortful self-control; CEI, cognitive effort investment; t_1_, before treatment; t_2_, after treatment.*

*For all coefficients *p* < 0.001 (Bonferroni-adjusted α = 0.0018).*

##### Abridged Cognitive Effort Scale (ACES; Kührt et al., 2021)

Need for Cognition and self-regulation (self- and effortful control) were measured with the Abridged Cognitive Effort Scale (ACES), a so far unpublished 24-item self-report scale that assesses an individuals’ propensity to invest cognitive effort, i.e., the motivational disposition to persistently engage in cognitively challenging tasks and situations and to maintain this motivation even against inner and outer obstructions. The scale was developed for research purposes and consists of four subscales with six items each of the following four well-established questionnaires: (1) the German version of the short NFC scale (Bless et al., 1994), (2) the German Intellect scale (Mussel, 2013), (3) the German adaptation of the short form of the Self-Control Scale (Bertrams and Dickhäuser, 2009), and (4) the effortful control scale of the German version of the Adult Temperament Questionnaire (Wiltink et al., 2006). Whereas (1) and (2) assess an individual’s cognitive motivation to enjoy and engage in intellectual achievements, (3) measures an individual’s capacity to overcome or alter emotional, cognitive, and behavioral responses, and (4) the ability to focus and shift attention. Participants are asked to indicate their agreement with statements such as “I really enjoy a task that involves coming up with new solutions to problems” or “I wish I had more self-discipline” on a 7-point rating scale from −3 = *strongly disagree* to +3 = *strongly agree*. For each of the four subscales (i.e., NFC, Intellect, Self-Control, Effortful Control), a sum score is calculated from the six items of the respective scales. Scale development was based on a previous investigation on the relationship between NFC and self-control (Kührt et al., 2021). For the ACES, the six items per scale with the highest item-total correlations were selected. In this study, a confirmatory factor analysis showed that the scale scores of the NFC- and intellect scales loaded on a common “cognitive motivation” (COM) factor and that the scale scores of the self- and effortful control scales loaded on an “effortful self-control” (ESC) factor. The primary factors, again, loaded on a higher order secondary factor “cognitive effort investment” (CEI). Higher scale scores represent enjoyment and persistence in thinking and problem solving against inner and outer resistance. The Cronbach’s alpha for both samples (COM at t_1_: 0.88 ≤α≤ 0.90; COM at t_2_: 0.76 ≤α≤ 0.77; ESC at t_1_: α = 0.89; ESC at t_2_: α = 0.90) are reported in Table 1 and are comparable to those of the NFC and effortful control scales observed by Nishiguchi et al. (2016).

#### Statistical Analyses

All analyses were performed using RStudio (Version 1.2.5019; RStudio Team, 2019) with R 3.6.1 (R Core Team, 2019) and the additional packages *psych* (Version 1.9.12.31; Revelle, 2019), *car* (Version 3.0-8; Fox and Weisberg, 2019), *semPlot* (Version 1.1.2; Epskamp, 2019), *shape* (Version 1.4.4; Soetaert, 2018), *boot* (Version 1.3-25; Davison and Hinkley, 1997; Canty and Ripley, 2020) and *lavaan* (Version 0.6-6; Rosseel, 2012). Prior to the analyses, the total sample was randomly split into two (sub)samples. Thereby, any effects that might have appeared due to different times and capacity utilization during data collection were controlled. This procedure further allowed to initially test and subsequently replicate the assumed models and effects economically and with enough statistical power as the sizes of both samples still exceeded the required sample size of 250 for achieving stable estimates for correlations (Schönbrodt and Perugini, 2013).

All analyses targeting at NFC and self-regulation were performed at the latent construct level: NFC was assessed by means of the primary factor COM, self-regulation by means of the primary factor ESC. In order to test the assumed cross-sectional effects, descriptive and bivariate correlational analyses for all t_1_- and t_2_-scores were initially performed. Due to mostly non-normal distributions of the parameters, Spearman’s Rank coefficients were computed.

**FIGURE 1:**
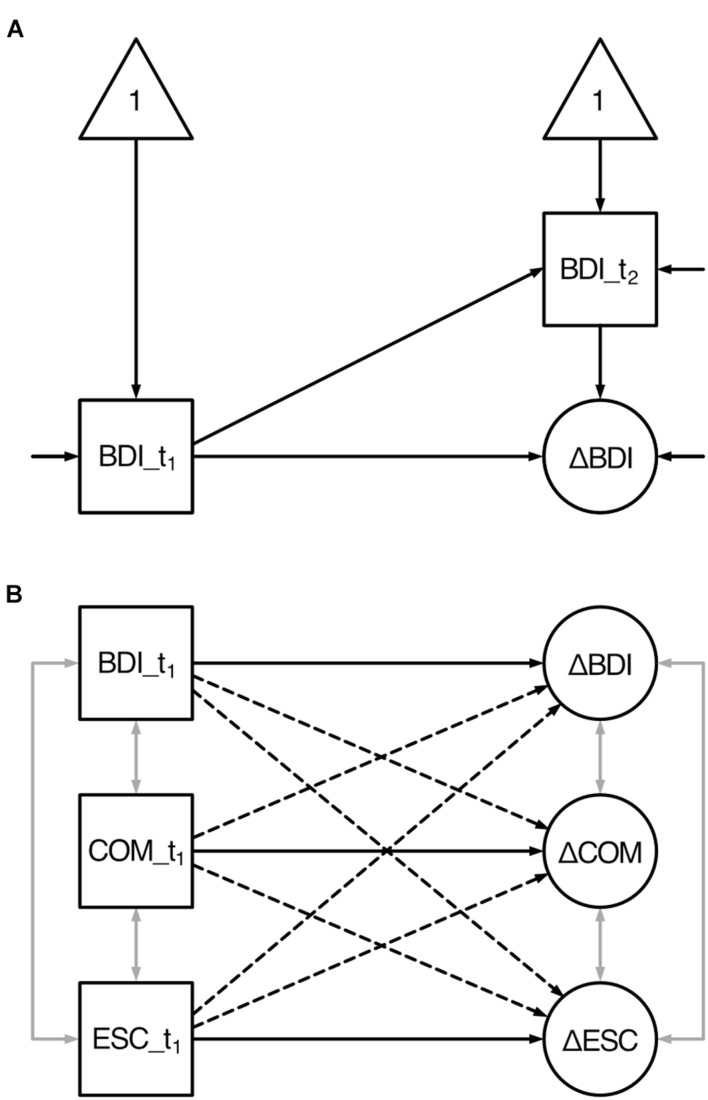
Latent change score model of the hypothesized longitudinal effects between all variables’ baseline and change scores. BDI, depressive symptoms; COM, cognitive motivation; ESC, effortful self-control. t_1_ denotes to the first assessment (baseline); t_2_ denotes to the second assessment; Δ denotes to the latent change score. Squares indicate observed variables; ellipses indicate latent variables; triangles indicate constants (intercepts). One-sided arrows indicate directed effects (regression coefficients) except for those without origin (variances), two-sided gray arrows indicate undirected relationships (covariances). **(A)** Univariate latent change score model exemplified by depressive symptoms. Depressive symptoms are assessed at two time points (BDI_t_1_ and BDI_t_2_). The change (ΔBDI) between the two assessments is modeled as latent variable. **(B)** Simplified model (without t_2_ observed variables, intercepts, and variances) illustrating the longitudinal effects: The change score of each variable is predicted by its corresponding baseline scores (i.e., self-feedback indicated by solid lines) as well as the baseline scores of the two remaining variables (i.e., cross-domain coupling indicated by dashed lines).

In order to test the assumed longitudinal effects, we established a model that allowed to examine the associations between the t_1_-scores and the change scores of all variables by using latent change score modeling (see Kievit et al., 2018). Representing a class of structural equation models, this approach seemed particularly suitable as we intended to explore the changes in the latent variables cognitive motivation (COM), self-regulation (ESC), and depressive symptoms (BDI). By providing an index of change (a latent change score, LCS) that expresses the average change in the scores from one time point (e.g., t_1_) to another (e.g., t_2_), this model provided information about (a) whether there were real changes in the scores from t_1_ to t_2_ (i.e., the LCS), (b) the extent to which individuals differed in these changes, and (c) the magnitude to which these changes related to t_1_-scores (Kievit et al., 2018). Thereby cross-domain effects, i.e., whether the change in one domain (e.g., self-regulation) is a function of the baseline score of another (e.g., depressive symptoms), which are referred to as cross-domain coupling (Kievit et al., 2018), could be examined. This model (see Figure 1) thus included the LCS of all variable domains (ΔCOM, ΔBDI, and ΔESC), each of which was measured by the respective scores at t_2_ regressed on the scores at t_1_. The LCS for each domain were then regressed on the scores at t_1_, both within each domain (self-feedback) and across domains (cross-domain coupling). The model further allowed to examine the associations between the t_1_- as well as between the change scores (i.e., covariances). In order to account for potential violations of the required assumptions (e.g., multivariate normal distribution, homoscedasticity), robust maximum likelihood estimations (MLR) were used, which allowed estimating robust (Huber-White) standard errors as well as scaled chi-square test statistics (Rosseel and Jorgensen, 2020).

### Results

#### Cross-Sectional Analyses

Results of the descriptive and correlational analyses are summarized in Table 1. All correlations were highly significant (*p* < 0.001, Bonferroni-adjusted α = 0.0018) and in the expected direction thereby confirming our assumptions: While COM was strongly positively associated with ESC, BDI was strongly negatively related to both, COM as well as ESC, before and after treatment.

#### Longitudinal Analyses

##### Latent Change Score Modeling

**FIGURE 2:**
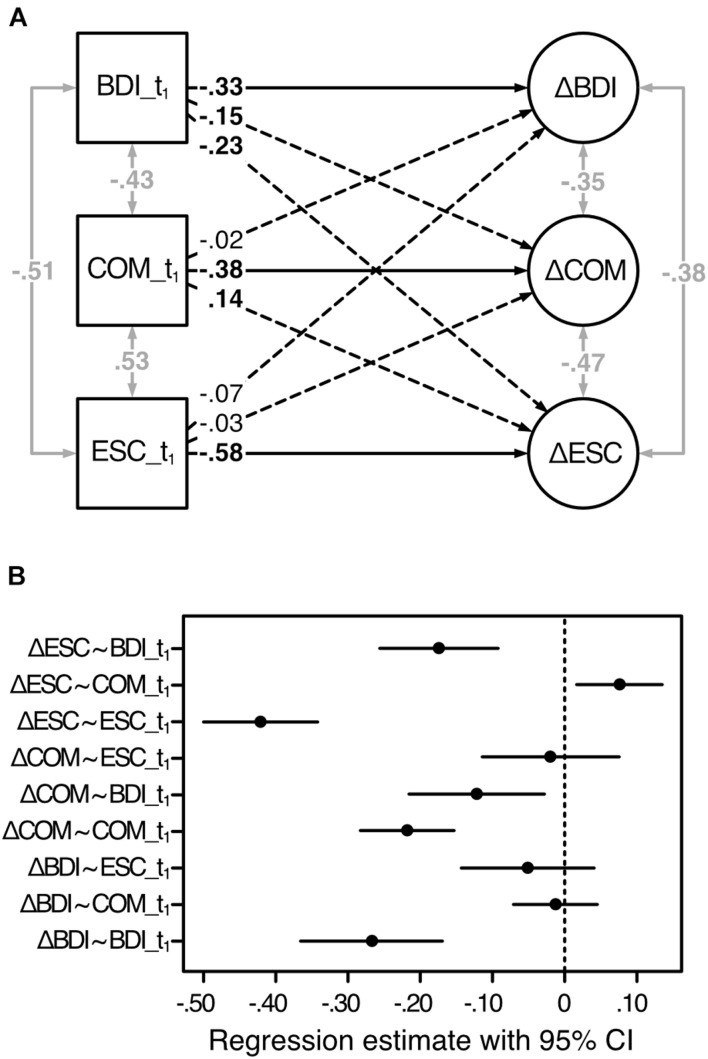
Estimated latent change score model of the change scores predicted by the baseline scores. Analysis based on the first sample (*n*_1_ = 459). BDI, depressive symptoms; COM, cognitive motivation; ESC, effortful self-control; t_1,_ before treatment; Δ, latent change score (i.e., the change from t_1_ to t_2_). **(A)** Standardized regression coefficients are displayed. One-sided solid and dashed arrows refer to the model in Figure 1 and indicate directed effects (regression coefficients), two-sided gray arrows indicate undirected relationships (correlations). Coefficients in bold are significant for *p* < 0.05. **(B)** 95% confidence intervals of the unstandardized regression estimates, based on 1,000 bootstrap samples.

The estimated latent change score model and standardized path coefficients are depicted in Figure 2A, confidence intervals (CI) of the unstandardized coefficients are shown in Figure 2B.

Supporting our hypothesis, the t_1_-scores of each variable significantly predicted their corresponding LCS: COM: *b* = −0.22, 95% CI (−0.28; –0.15), β = −0.38; ESC: *b* = −0.42, 95% CI (−0.50; −0.34), β = −0.58; BDI: *b* = −0.27, 95% CI (−0.37; −0.17), β = −0.33. As LCS can display increasing as well as decreasing scores from t_1_ to t_2_, negativity and positivity of the coefficients have to be interpreted separately for each variable and in the light of the magnitude of the baseline scores. Thus, while the negative associations between COM_t_1_ and ΔCOM as well as between ESC_t_1_ and ΔESC indicate that lower baseline scores were associated with stronger increases in COM, respectively, ESC from t_1_ to t_2_, the negative association between BDI_t_1_ and ΔBDI indicates that higher scores at t_1_ were associated with a stronger amelioration from t_1_ to t_2_. As expected, COM_t_1_ significantly positively predicted the change in ESC [*b* = 0.08, 95% CI (0.02; 0.14), β = 0.14].^2^ Higher baseline scores of COM thus predicted more available ESC resources at t_2_. Contrary to our assumptions, ESC_t_1_ did neither predict any change in COM [*b* = −0.02, 95% CI (−0.11; 0.07), β = −0.03] nor in BDI [*b* = −0.05, 95% CI (−0.14; 0.04), β = −0.07]. Rather unexpectedly, we found ΔCOM and ΔESC to be significantly negatively predicted by BDI_t_1_ [COM: *b* = −0.12, 95% CI (−0.22; −0.03), β = −0.15; ESC: *b* = −0.17, 95% CI (−0.26; −0.09), β = −0.23]. In both cases, the negativity of the coefficients indicated that lower BDI scores before treatment were associated with smaller releases of capacities in both, COM as well ESC, from t_1_ to t_2_.

##### Mediation Analysis

**FIGURE 3:**
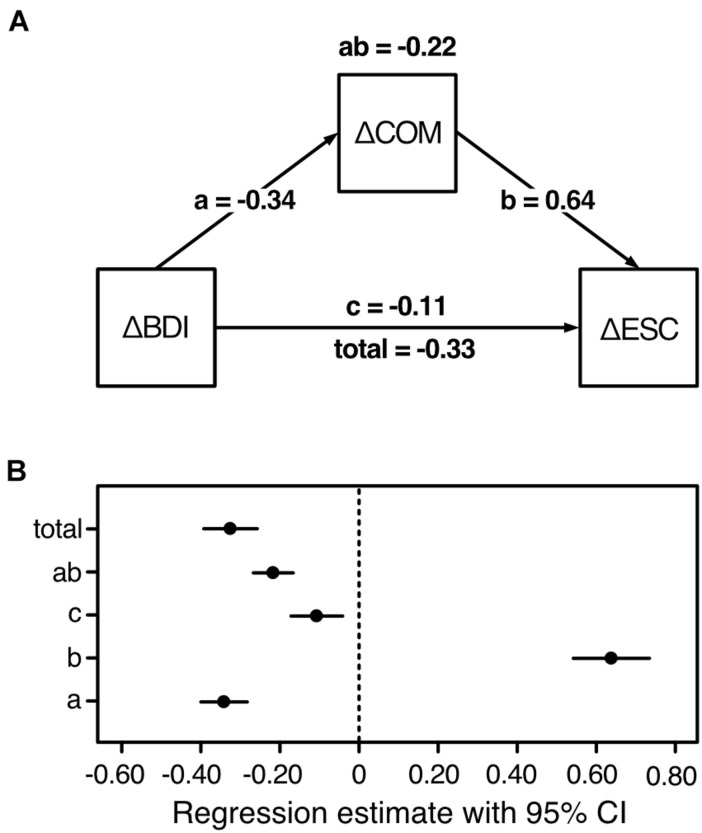
Results and model of the mediation analysis of the first sample (*n*_1_ = 459). BDI, depressive symptoms; COM, cognitive motivation; ESC, effortful self-control; Δ, latent change score (i.e., the change from t_1_ to t_2_). **(A)** Increasing cognitive motivation partially mediates the association between decreasing depressive symptoms and increasing self-regulation. The following unstandardized effects are displayed: total (direct effect without mediation) = ab (indirect mediating effect) + c (direct effect with mediator). **(B)** *95%* confidence intervals of the unstandardized regression estimates, based on 1,000 bootstrap samples.

The results of latent change score modeling suggested that (1) the change in self-regulation was partly driven not only by baseline depressive symptoms, but also by baseline cognitive motivation with the change in the latter also being a function of baseline depressive symptoms; and that (2) the change in all three variables was intercorrelated. Taken together with the finding of Nishiguchi et al. (2016) that cross-sectionally, cognitive motivation (NFC) partly mediated the relationship between depressive symptoms and self-regulation (effortful control), we explored whether the change in COM would mediate the relationship between the change in BDI and the change in ESC. To this end, the individual scores of the latent change factors were extracted and submitted to a mediation analysis. Model and unstandardized path coefficients are depicted in Figure 3A. Figure 3B shows the CI of the unstandardized regression estimates. The direct negative effect of ΔBDI on ΔESC [*b*_*total*_ = −0.33, 95% CI (−0.39; −0.26), β_*total*_ = −0.07] was reduced, but still significant, after ΔCOM had been considered as mediator [*b*_c_ = −0.11, 95% CI (−0.17; −0.04), β_c_ = −0.02].^3^ With a significant indirect effect of *b*_*ab*_ = −0.22, 95% CI (−0.27; −0.17), β_ab_ = −0.05, the present analysis thus revealed a partial mediating effect of ΔCOM. These findings indicate that the change in ESC was partly the direct result of the change in BDI scores and partly the indirect result of changing COM, which accompanied the change in BDI scores [*b*_a_ = −0.34, 95% CI (−0.40; −0.28), β_a_ = −0.08] and, due to its positive association with self-regulation, led to its change [*b*_b_ = 0.64, 95% CI (0.54; 0.73), β_b_ = 0.61].

### Discussion

The cross-sectional results of the first sample corresponded to earlier findings (Bertrams and Dickhäuser, 2012; Nishiguchi et al., 2016) as well as our assumption that cognitive motivation and self-regulation would be positively intercorrelated and negatively to depressive symptomatology both before and after treatment.

In order to investigate the assumed longitudinal effects, we analyzed a model that was conceptually based on Nishiguchi et al. (2016), but used latent change score modeling instead of path analysis. Expanding upon the findings of Nishiguchi et al. (2016) we found the baseline scores of all variables to significantly predict their corresponding change scores.

Contrary to former results (Nishiguchi et al., 2016) and our predictions, we did not find baseline self-regulation to predict any changes in cognitive motivation or depressive symptomatology. Yet, Nichiguchi et al. examined individuals with subclinical depression, who might have had enough self-regulating capacities left that could be directed into changing motivational efforts or depressive symptoms. Our findings are based on a clinical sample of individuals who suffered from severe functional impairments that required psychosomatic rehabilitation treatment. The present finding nevertheless to some extent resonated with our presented line of reasoning. As we have argued, the majority of resources pertaining to effortful self-control, i.e., adaptive self-regulation, at baseline may have been bound by the depressive state and thus directed to maladaptive responses. Therefore, before treatment, not enough capacities may have been available that could have affected changing motivational or depression scores. Rather, at first, more (cognitive motivational) resources needed to be released in order to “boost” (Bertrams and Dickhäuser, 2012; p. 71) and/or, re-direct the few but existing self-regulating capacities toward a more functional realignment of attention.

In line with this, we found the change in self-regulation to be significantly predicted by baseline depression scores, which was further predictive for the change in cognitive motivation. Finally, in line with our assumption, the change in self-regulation was significantly predicted by baseline cognitive motivation in that higher baseline motivation was associated with stronger changes in self-regulation. This data pattern suggests that depressive symptoms at first assessment might determine the characteristics as well as changes of the capacities that might be utilized by self-regulation and cognitive motivation rather than the other way round.

Based on these findings, we conducted a mediation analysis, which revealed a partial mediating effect of the change in cognitive motivation on the negative association between the changes in depressive symptoms and effortful self-control. This finding might indicate that when depressive symptomatology decreased from baseline to second assessment, this may have set capacities free. This not only directly led to more available self-regulation capacities, but also indirectly *via* simultaneously releasing resources pertaining to cognitive motivation, which further benefitted self-regulation. Our results thus expand the cross-sectional mediation model that was proposed by Nishiguchi et al. (2016), and suggest that cognitive motivation might be actively involved in the longterm association between depressive symptoms and self-regulation.

In order to confirm and evaluate these findings, particularly the mediating role of cognitive motivation as well as the longitudinal effects of depressive symptoms on cognitive motivation and self-regulation, we repeated all analyses within the second sample. Based on our findings, we cross-sectionally assumed, that (1) cognitive motivation and self-regulation would be positively associated and that (2) depressive symptoms would be negatively associated with cognitive motivation as well as self-regulation. On the basis of the longitudinal results and the proposed model (Figure 1B), we assumed that (3) baseline depressive symptoms, cognitive motivation, and self-regulation would predict its corresponding change, and that further (4) baseline cognitive motivation would predict the change in self-regulation, and that (5) baseline depressive symptoms would predict the changes in cognitive motivation and self-regulation. As mechanism underlying the association between the changes in depressive symptoms and self-regulation, we (6) expected to find the change in cognitive motivation to at least partially mediate the association between the changes in depressive symptoms and self-regulation.

## Sample 2: Crossvalidation/Test of the Model

### Materials and Methods

All data, analyzing scripts and materials for reproducing the analyses of the second sample are permanently and openly accessible at the OSF website mentioned above. The analyses of this study were pre-registered at https://aspredicted.org/mg4sg.pdf.

#### Participants and Procedure

Analyses of the second sample included *n*_2_ = 468 individuals (68.59% females) as 42 data sets of the original 530 had to be excluded due to missing values, and 20 due to sub-threshold BDI-II scores as only complete cases with baseline BDI-II scores above 8 were included in the present analyses.^4^ As in study 1, patients of the second sample (if at all) only received antidepressant medication in accordance with the guidelines for rehabilitation treatment. Mean age of this sample was 51.54 years (*SD* = 8.92). First and second assessment was conducted within a median time distance of 33 days (range_*ACES*_: 14 to 75 days) and 33.5 days (range_*BDI–II*_: 14 to 75 days), respectively. In order to examine, whether the two samples differed with respect to sample characteristics as well as the main variables, unequal variance *t*-tests were performed (Ruxton, 2006). No differences were found with respect to age [*t*_(923.72)_ = 0.89, *p* = 0.376], gender [*t*_(924.27)_ = −0.34, *p* = 0.732], nor any of the variables’ means: COM_t_1_ [*t*_(916.48)_ = −0.33, *p* = 0.739], COM_t_2_ [*t*_(920.71)_ = 0.22, *p* = 0.826], ESC_t_1_ [*t*_(924.53)_ = 0.36, *p* = 0.721], ESC_t_2_ [*t*_(924.73)_ = 0.33, *p* = 0.742], BDI_t_1_ [*t*_(922.05)_ = 0.22, *p* = 0.826], BDI_t_2_ [*t*_(922.95)_ = 0.72, *p* = 0.471].

#### Measures

The same measures and procedure as described for sample 1 were applied. For Cronbach’s alpha of the applied scales see Table 1. Majority of the scales showed satisfactory to excellent internal consistencies (0.77 ≤α≤0.94) and thereby confirmed those observed in the first sample.

#### Statistical Analyses

All analyses were run according to the pre-registered procedure with RStudio and the packages described above. All analyses were performed at the latent construct level (i.e., COM, ESC, and BDI). In order to test hypotheses (1) and (2), Spearman’s Rank coefficients were computed between all t_1_- and t_2_-variable scores as not all parameters were normally distributed. For the longitudinal hypotheses (3), (4), and (5), the established model (Figure 1) was analyzed using latent change score modeling. In order to test the proposed mediation model (hypothesis 6), the individual scores of the latent change factors were used for mediation analysis with *lavaan*. Indirect effects were analyzed according to the first sample and robust maximum likelihood estimations (MLR) were used.

### Results

#### Cross-Sectional Analyses

Results of the descriptive and correlation analyses are summarized in Table 1. All coefficients were highly significant (*p* < 0.001, Bonferroni-adjusted α = 0.0018). As predicted, COM and ESC were positively associated, and BDI was negatively related to COM as well as ESC, before and after treatment.

#### Longitudinal Analyses

##### Latent Change Score Modeling

**FIGURE 4:**
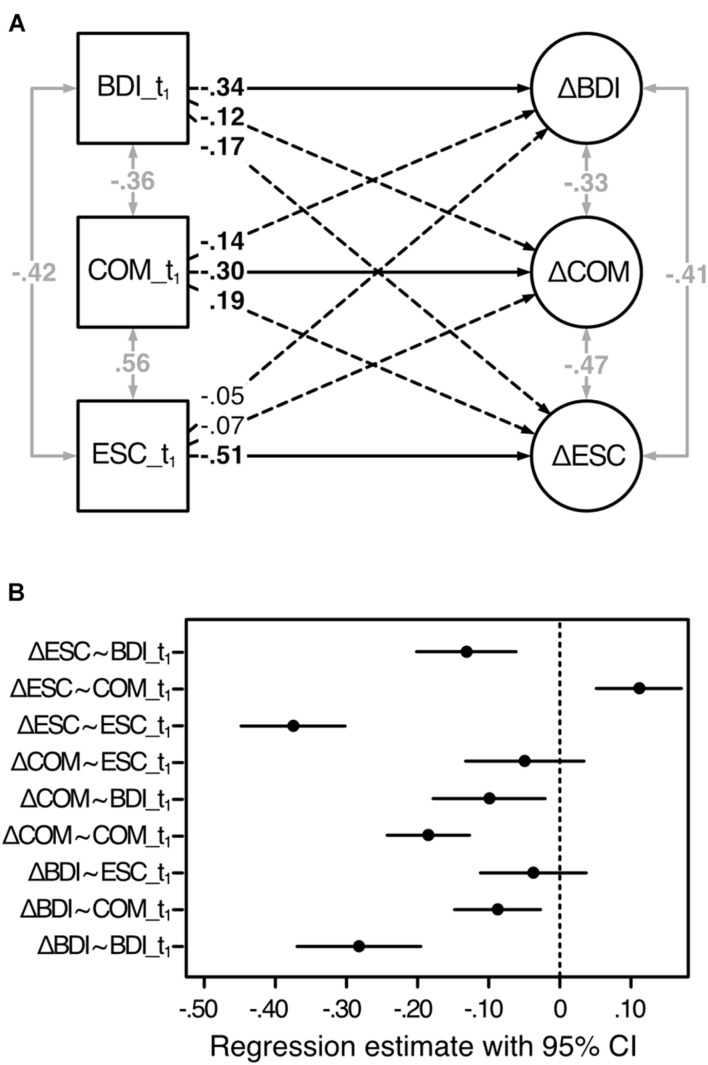
Estimated latent change score model of the change scores predicted by the baseline scores. Analysis based on the second sample (*n*_2_ = 468). BDI, depressive symptoms; COM, cognitive motivation; ESC, effortful self-control; t_1,_ before treatment; Δ, latent change score (i.e., the change from t_1_ to t_2_). **(A)** Standardized regression coefficients are displayed. One-sided solid and dashed lines refer to the model in Figure 1 and indicate directed effects (regression coefficients), two-sided gray arrows indicate undirected relationships (correlations). Coefficients in bold are significant for *p* < 0.05. **(B)** 95% confidence intervals of the unstandardized regression estimates, based on 1,000 bootstrap samples.

Estimated latent change score model and standardized path coefficients are depicted in Figure 4A, CI of the unstandardized coefficients are shown in Figure 4B.

As hypothesized, the change in each variable was significantly predicted by its corresponding t_1_-scores: COM: *b* = −0.18, 95% CI (−0.24; −0.13), β = −0.30; ESC: *b* = −0.38, 95% CI (−0.45; −0.30), β = −0.51; BDI: *b* = −0.28, 95% CI (−0.37; −0.20), β = −0.34. Thus, whereas lower baseline scores of COM and ESC predicted stronger changes in COM and ESC, respectively, higher baseline BDI scores predicted stronger changes in BDI from t_1_ to t_2_. Our assumption, according to which COM_t_1_ would significantly predict the change in ESC, was also confirmed [*b* = 0.11, 95% CI (0.05; 0.17), β = 0.19]. Rather unexpectedly, though, we found COM_t_1_ to further significantly negatively predict ΔBDI [*b* = −0.09, 95% CI (−0.15; −0.03), β = −0.14] indicating that higher baseline COM predicted a stronger release of resources from t_1_ to t_2_. As expected, ΔCOM and ΔESC were significantly negatively predicted by BDI_t_1_ [COM: *b* = −0.10, 95% CI (−0.18; −0.02), β = −0.12; ESC: *b* = −0.13, 95% CI (−0.20; −0.06), β = −0.17] indicating that lower depression scores before treatment predicted only small changes in available COM as well ESC capacities from t_1_ to t_2_.

##### Mediation Analysis

**FIGURE 5:**
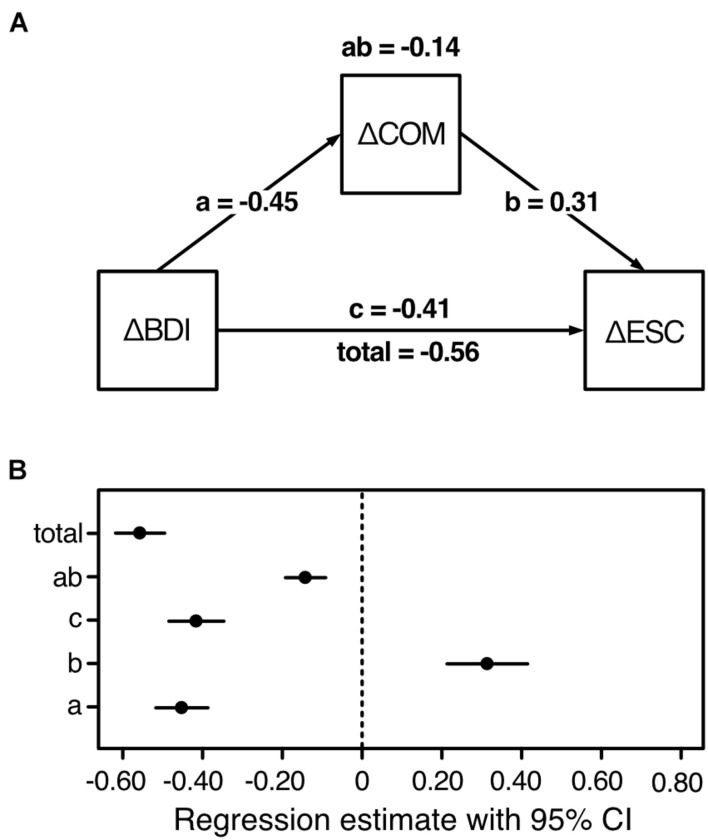
Results and model of the mediation analysis in the second sample (*n*_2_ = 468). BDI, depressive symptoms; COM, cognitive motivation; ESC, effortful self-control; Δ, latent change score (i.e., the change from t_1_ to t_2_). **(A)** Increasing cognitive motivation partially mediates the association between decreasing depressive symptoms and increasing self-regulation. The following unstandardized effects are displayed: total (direct effect without mediation) = ab (indirect mediating effect) + c (direct effect with mediator). **(B)** *95%* confidence intervals of the unstandardized regression estimates, based on 1,000 bootstrap samples.

Model, unstandardized path coefficients, and CI of the unstandardized regression estimates are depicted in Figures 5A,B. The results confirmed our hypothesis. The total effect of ΔBDI on ΔESC [*b*_*total*_ = −0.56, 95% CI (−0.62; −0.49), β_*total*_ = −0.11] was significantly, albeit not completely, reduced when ΔCOM was included as mediator [*b*_*c*_ = −0.41, 95% CI (−0.48; −0.34), β_*c*_ = −0.08]. This was reflected in the significant indirect effect [*b*_*ab*_ = −0.14, 95% CI (−0.19; −0.09), β_*ab*_ = −0.03]. The findings thus indicate that the change (i.e., increasing capacities) in COM, which was negatively associated with the change (i.e., release of capacities) in BDI [*b*_*a*_ = −0.45, 95% CI (−0.52; −0.39), β_*a*_ = −0.10] and positively with the change (i.e., availability of resources for) in ESC [*b*_*b*_ = 0.31, 95% CI (0.21; 0.42), β_*b*_ = 0.29], partially mediated the association between decreasing resources bound by depressive symptomatology and increasing resources available for self-regulation.

### Discussion

The analyses of the second sample replicated and substantiated the findings of the first sample. With respect to our cross-sectional assumptions, we found cognitive motivation and self-regulation to be strongly positively interrelated, and depressive symptoms to show a strong, but inverse relationship to both aspects at both times of assessment. The results of sample 2 showed a nearly identical pattern as observed for sample 1, and thereby robustly demonstrate the validity of equivalent previous findings (Bertrams and Dickhäuser, 2012; Nishiguchi et al., 2016) for the clinical setting.

The longitudinal results of the second sample equally confirmed the first sample’s findings and thus the hypotheses derived from them, thereby anew expanding upon the reports by Nishiguchi et al. (2016): Not only did we find the baseline scores of all variables to predict their corresponding latent change scores, we also found the change in self-regulation to be predicted by baseline cognitive motivation as well as baseline depressive symptoms. We further found baseline depression scores to predict the change in cognitive motivation.

Rather unexpectedly, we found baseline cognitive motivation to additionally predict the change in depressive symptomatology. This finding needs verification: if replicable it would suggest a more decisive role of cognitive motivation within the underlying resource (re-)allocation process than previously assumed. Yet, confirming our expectation, we found that increasing the resources available for cognitive motivational processes partially mediated the relationship between decreasing resources tied to depressive symptomatology and increasing self-regulation capacities.

The analyses of the second sample thus confirmed the robustness of the first sample’s findings. Beyond that, they added substantially to previous reports on the associations between depressive symptoms, cognitive motivation, and self-regulation and point to cognitive motivation as a logically and statistically independent, co-determining factor in the re-allocation and enhancement of self-regulating capacities in the course of treatment in a clinical sample.

## General Discussion

With the present study we aimed to investigate the cross-sectional and longitudinal associations between cognitive motivation, self-regulation, and depressive symptoms in in-patients of psychosomatic rehabilitation before and after treatment. Extending previous findings, we established a model that incorporated latent change instead of purely cross-sectional scores and analyzed it in a large clinical sample.

Cross-sectionally, we found self-regulation and cognitive motivation to be strongly positively related to each other and strongly negatively to depressive symptoms. Whilst the observed positive association between cognitive motivation and self-regulation extends the relevance of corresponding earlier findings for the first time to the clinical setting, the negative relations between depressive symptoms and cognitive motivation as well as self-regulation might be taken to genuinely reflect the cognitive deficits that have been shown to accompany depression (e.g., Gotlib and Joormann, 2010).

Following the idea of limited attentional capacity and altered resource allocation in depression (e.g., Ellis and Ashbrook, 1988; Levens et al., 2009), we found that higher baseline depression scores predicted stronger symptom decreases (and thus resource releases) from the first to the second assessment, and that lower initial scores in cognitive motivation and self-regulation were predictive for stronger resource increases of the same. These results expand upon the findings of Nishiguchi et al. (2016): By incorporating latent change instead of the second assessments’ scores, our longitudinal model was able to simultaneously and coherently explore the associations between the baseline and the (latent) change scores as well as the resource (re)allocation mechanism that was assumed to underlie the observed interdependencies.

In line with our model, we found the change in self-regulation to be significantly predicted by baseline cognitive motivation and depression scores, the latter of which further being predictive for the change in cognitive motivation. So, the present analysis was able to convincingly reveal the predictive effects of baseline and changing depressive symptomatology in one step: The results of both samples demonstrate that it is indeed the depressive state before treatment that determines the allocation of resources to effortful processes such as self-regulation and cognitive motivation rather than the other way round. Thus, whilst depressive symptoms might have affected the allocation of resources of cognitive motivation and self-regulation before treatment, with symptoms and corresponding resource demands decreasing over the course of treatment, the formerly tied resources might have subsequently become allocable again. This is reflected in the decrease of depressive symptoms (and the associated resource release) and the corresponding increase of distributable cognitive motivational and self-regulating capacities from the first to the second assessment. Our findings thereby prove that aversive affective states inhere a crucial role in the allocation and availability of attentional resources (Ellis and Ashbrook, 1988).

The present results further indicate a fundamental role of cognitive motivation in resource allocation and to the benefit of self-regulation. As mechanism that might underlie the observed positive association between these effortful processes, Bertrams and Dickhäuser (2012) suggested that both might rely on the same (energy) resource and that regular cognitive engagement might “boost” (p. 71) executive resources. In line with this, the authors suggested a mediation model, in which self-regulation (self-control) completely mediated the association between cognitive motivation (NFC) and affective adjustment (e.g., depressed mood). Our data, however, did not support this model as we did not find baseline self-regulation to predict any change in cognitive motivation or depressive symptoms. In fact, our results are in line with previous reports on compromised cognitive and particularly self-regulating capacities in depression (Tice, 2009; Gotlib and Joormann, 2010; Krämer et al., 2014). In the present context, our findings might particularly illustrate that self-regulating capacities were indeed bound by the regulation of the emotional aversive mood state at first assessment, so that no self-regulating reserves might have been left that could be allocated (to cognitive motivation) or invested to “initiate” any change (in depressive symptoms). Our finding that the association between the changes in depressive symptoms and self-regulation was partially mediated by the change in cognitive motivation thereby in principle corresponds to the mediation mechanism proposed by Nishiguchi et al. (2016). By incorporating longitudinal data, our model, however, was able to support the assumed resource (re-)allocation process.

The observed (co-)determining effect of cognitive motivation was further corroborated by the unexpected finding in sample 2, that baseline cognitive motivation scores predicted the change in depressive symptoms. Being aware of the preliminary nature of this finding, this finding might lead to suggest that the resources pertaining to cognitive motivation might not have been totally exhausted before treatment. Having sufficient resources left, individuals with higher cognitive motivation might, in consequence, have been more capable of allocating cognitive effort not only to the engagement in more adaptive self-regulating behavior, but also to the disengagement from negative stimuli. This would support previous reports according to which not only emotional states but also (cognitive) motivation affect the (re)allocation of cognitive and particularly attentional resources (Ellis and Ashbrook, 1988; Enge et al., 2008; Pessoa, 2009), and could suggest that multiple pathways may underlie and converge to the enhancement of executive attention and thus self-regulation.

The partly deviating findings of Nishiguchi et al. (2016) might to a certain extent be explained by the different analyzing procedures, but rather by the different sample composition: Nishiguchi et al. (2016) investigated a non-clinical sample, of which only a fifth showed clinically relevant depressive symptoms. The detrimental effects of depressive symptoms on self-regulation and corresponding resource allocation might have been too small and not significant enough to completely incapacitate self-regulation, which might thus have been sufficiently pronounced to determine depressive symptoms and cognitive motivation at second assessment. As our analyses were based on individuals with clinically relevant depression scores, our findings might therefore not contradict these findings, but rather illustrate how these aspects are developed and interrelated when corresponding capacities are substantially compromised. Referring to our line of argumentation, aversive affective states may thus have to exceed a particular (clinical) threshold in order to affect the amount and availability of resources. However, when the affective distortions exceed the clinical threshold, as might notably be the case when individuals suffering from their symptoms seek professional treatment, these mood states might even be able to change or interfere with executive capacities that pertain to relatively stable individual dispositions such as cognitive motivation and self-regulation. In this respect, Costa et al. (2005), however, argued that such changes might not reflect “state artifacts of the depressed mood but genuine changes in the biological bases underlying the traits themselves” (p. 46).

### Limitations and Future Research

This study has some limitations that should be noted. All variables of interest were assessed by means of well-validated self-report questionnaires. Global retrospective self-reports on negative affective states, such as required by the depression inventory used here, on the one hand, are prone to selective (memory and negative) bias, which can cause overestimation of symptoms (Sato and Kawahara, 2011). Personality questionnaires, such as the applied ACES, on the other hand, may have to deal with social desirability and acquiescence, which can affect the factor structure as well as the association between items and with other variables (Danner et al., 2015; Navarro-González et al., 2016). For the same reasons and beyond, it has been debated whether personality traits can be validly assessed at all during (acute) mental states (Costa et al., 2005). In this respect, Costa et al. (2005), however, argued that personality traits might not *per se* be understood as immune to change, and in the context of psychiatric disorders might reflect alterations in the underlying biological and physiological mechanisms rather than invalid assessment. This corresponds to the rationale of the present study. As, however, the present sample mainly consisted of patients with depressive symptoms, selective attention, limited concentration as well as attempts to meet assumed social expectations (e.g., of the therapists) might have compromised response quality.

By using depressive symptoms as key variable, this study allowed to directly compare the present results with previous findings (Nishiguchi et al., 2016). It is of note, though, that participants of the present samples were heterogeneous in terms of their depressive syndrome. As the severity of the symptoms did not always justify the assignment of depression as a primary diagnosis, depression could also be the secondary and thus comorbid diagnose if other symptoms predominated. Thus, in order to confine the findings to depression, future studies should focus on individuals who are primarily diagnosed with depression and exclude or control for comorbidity.

Furthermore, the medication status has not been considered in the present analyses. However, medication might influence cognitive functioning in various ways and individuals taking medication might differ from drug-naïve individuals (e.g., Tancer et al., 1990). However, as outlined above, no psychotropic medication was administered. Antidepressant medication was administered only in some patients and in accordance with the guidelines for rehabilitation treatment. Nevertheless, future studies should assess and control for medication as well as corresponding changes between the first and second assessment.

Finally, although the interval between the first and the second assessment met earlier reports (Nishiguchi et al., 2016), it might have been rather short. As assessment times were tied to the standard length of stay in rehabilitation (Linden, 2014), which cannot be expanded, future studies should incorporate a follow-up assessment after discharge of the patients. This would allow to estimate the long-term stability of favorable outcomes and to investigate the development of the variables of interest here after and independent of treatment.

### Conclusion and Implications

The present study enriches the current state of research by illustrating how depressive symptoms interact with effortful cognitive processes in a sample of in-patients during psychosomatic rehabilitation. Altogether our findings indicate that resource (re-)allocation might constitute the crucial mechanism by which depressive symptoms impact on effortful cognitive processes and particularly how they allocate corresponding resources. At the same time, this mechanism might represent a target point for limiting the intrusive effects of depression by strengthening those capacities that are involved in resource allocation. The present results point to cognitive motivation as such a personal capacity that could not only favor the change in self-regulation as a consequence of decreasing depressive symptomatology, but rather serve as an independent and at least partial catalyst for this change.

As in other settings, psychosomatic rehabilitation primarily aims at decreasing symptoms and regaining functionality (Linden, 2014). As depressive symptoms may, however, chronify or reoccur (Klein and Allmann, 2014; Köllner, 2016), treatments may additionally focus on strengthening affected as well as potential compensating capacities (Linden, 2014). Our findings suggest that cognitive motivation and self-regulation are promising candidates for this.

As self-regulation benefits from regular training (Baumeister et al., 2006), corresponding exercises might not only target at apparent behavior, but rather at the mechanisms that are assumed to underlie characteristic deficits (Siegle et al., 2007; Joormann and Gotlib, 2010). In this respect, a number of studies has found attentional training to effectively reduce attentional bias as well as depressive symptomatology (e.g., rumination) in both, non-clinical (Wells and Beevers, 2010; Yang et al., 2015) as well as clinical samples (Siegle et al., 2007; Beevers et al., 2015). Trainings in effortful (attentional) control and hence in the ability to appropriately allocate corresponding resources might therefore become a basic feature in the treatment of depressive symptoms and even precede interventions that target at more overt depression-related behavior and cognitions (Koster et al., 2011; Kanske and Kotz, 2012; Hoyer et al., 2020).

The identification of cognitive motivation as partial mediator between depressive symptoms and self-regulation might suggest an alternative starting point. By assessing cognitive motivation before treatment, interventions could be individually tailored in order to provide adequate support for regaining not only behavioral but also cognitive engagement. As cognitive motivation has notably been found to positively relate to adaptive forms of coping, self- and emotion regulation (Bye and Pushkar, 2009; Bertrams and Dickhäuser, 2012; Grass et al., 2018), promoting this motivational disposition might provide patients with the capacities that are essential for affective adjustment and mental health. Corresponding exercises could be coherently integrated into activity scheduling or (non-)social skills training in the context of BA (Kanter et al., 2010). Encouraging patients to engage in effortful but achievable cognitive endeavors could give them “a sense of control or mastery” (Osberg, 1987, p. 443) and thereby strengthen those capacities that are indispensable for adaptive self- and emotion regulation.

## Data Availability Statement

The datasets presented in this study can be found in online repositories. The names of the repository/repositories and accession number(s) can be found below: The original dataset of this study contained further demographic and treatment-related variables beyond those described here. As these were, however, either confidential or not relevant for the present study, these variables were removed. The datasets including those variables necessary for reproducing the presented analyses and results are permanently and publicly available at https://osf.io/e8tg5 (Strobel et al., 2020).

## Ethics Statement

The studies involving human participants were reviewed and approved by Ethics Committee of the Faculty of Behavioral and Social Sciences, Chemnitz University of Technology. The patients/participants provided their written informed consent to participate in this study.

## Author Contributions

AnS, AlS, and JH conceived and designed the study. VK and UM organized and supervised the data collection. AlS and AF analyzed the data. AF, AlS, and AnS wrote parts of the manuscript. AlS and AnS handled the revision of the manuscript. All authors gave the final approval of the manuscript to be published.

## Conflict of Interest

The authors declare that the research was conducted in the absence of any commercial or financial relationships that could be construed as a potential conflict of interest.

## Publisher’s Note

All claims expressed in this article are solely those of the authors and do not necessarily represent those of their affiliated organizations, or those of the publisher, the editors and the reviewers. Any product that may be evaluated in this article, or claim that may be made by its manufacturer, is not guaranteed or endorsed by the publisher.

## Footnotes

1 In addition to this manual-based cut-off, all analyses were repeated with (a) all individuals irrespective of their baseline BDI-II scores in order to account for the dimensionality of the construct, and (b) individuals with baseline BDI-II scores > 11 as this threshold has been shown to guide clinical practice (Riedel et al., 2010). The results of these analyses are provided in the supplementary material. Deviating results are mentioned in the corresponding result section.

2 Latent change score analyses including only individuals with baseline BDI-II scores > 11 did not find COM_t_1_ to significantly predict ΔESC [*b* = 0.06, 95% CI (−0.002; 0.12), β = 0.11]. The complete results are included in the supplementary material. The remaining results were essentially the same.

3 Mediation analyses including all individuals irrespective of the baseline BDI-II scores revealed a complete mediating effect: [*b*_*total*_ = −0.24, 95% CI (−0.32; −0.16), β_*total*_ = −0.05] = [*b*_c_ = −0.06, 95% CI (−0.13; 0.01), β_c_ = −0.01] + [*b*_*ab*_ = −0.18, 95% CI (−0.23; −0.13), β_*ab*_ = −0.04]. The complete results are included in the supplementary material. The remaining results were essentially the same.

4 In addition to the pre-registered procedure, all analyses were repeated as described in footnote 1. Results of these analyses are provided in the supplementary material. Deviating results are mentioned in the corresponding result section.
