# Supplementary material for: Cognitive Motivation as a Resource for Affective Adjustment and Mental Health
Source: Front Psychol. 2021 Sep 21;12:581681. doi: 10.3389/fpsyg.2021.581681 (PMC8490806; doi:10.3389/fpsyg.2021.581681)
Supplement: Supplementary file 1 [file Data_Sheet_1.PDF]

Cognitive motivation – a resource for affective adjustment & mental health (#39710)

Created: 04/22/2020 12:21 PM (PT)
Public: 07/07/2020 04:21 AM (PT)

Author(s)
Aniko Steiger (Chemnitz University of Technology) - aniko.steiger@psychologie.tu-chemnitz.de
Alexander Strobel (Technische Universität Dresden) - alexander.strobel@tu-dresden.de
Anja Strobel (Chemnitz University of Technology) - anja.strobel@psychologie.tu-chemnitz.de

1) Have any data been collected for this study already?
It's complicated. We have already collected some data but explain in Question 8 why readers may consider this a valid pre-registration nevertheless.

2) What's the main question being asked or hypothesis being tested in this study?
Cognitive motivation has been found to positively relate to self- and effortful control, and inversely to subclinical depression (e.g., Bertrams & Dickhäuser, 2012; Nishiguchi et al., 2016). As these findings are based on healthy samples and mostly on cross-sectional data, this study's primary aim was to investigate the longitudinal associations of cognitive motivation, self-regulation (self- and effortful control), and depressive symptoms as well as the potential mechanisms that may underlie these associations in a clinical sample before and after treatment.
Cross-sectionally we assume that (H1) cognitive motivation and self-regulation would be positively associated and (H2) depressive symptoms would be negatively associated with cognitive motivation as well as self-regulation.
Longitudinally we assume that (H3) baseline depressive symptoms, cognitive motivation, and self-regulation would predict its corresponding change, and that further (H4) baseline cognitive motivation would predict the change in self-regulation, and that (H5) baseline depressive symptoms would predict the changes in cognitive motivation and self-regulation. As mechanism underlying the association between the changes in depressive symptoms and self-regulation, we (H6) expect to find an at least partially mediating effect of the change in cognitive motivation.

3) Describe the key dependent variable(s) specifying how they will be measured.
The key dependent variables in H1 and H2 are the pre-(t1) and post(t2)-scores of cognitive motivation (COM; ACES, Kührt et al., 2019), self-regulation (ESC; ACES, Kührt et al., 2019), and depression (BDI; BDI-II, German version by Hautzinger et al., 2009).
The key dependent variables in H3, H4, and H5 are the latent change scores of COM, ESC, and BDI. Latent change scores are calculated by regressing the t2-score of each variable on its corresponding t1-score (Kievit et al., 2018). Afterwards, each change score is computed by means of its t2-score with a factor loading fixed to 1 (Kievit et al. 2018).
The key dependent variable in H6 is the ESC latent change score.

4) How many and which conditions will participants be assigned to?
There were no experimental conditions the participants were assigned to.

5) Specify exactly which analyses you will conduct to examine the main question/hypothesis.
H1 and H2 will be tested by means of correlational analyses. Therefore, t1- and t2-scores of BDI, COM, and ESC will be used.
The longitudinal effects stated in H3, H4, and H5 will be analyzed by using latent change score modeling according to Kievit et al. (2018). In the present model, the change in each variable (COM, ESC, BDI) will be regressed on the t1-scores of all variables. This allows to investigate cross-domain effects, i.e., whether the change in one domain (e.g., affect measured by BDI) is a function of the baseline score of another (e.g., motivation assessed by COM or cognition assessed by ESC) (Kievit et al., 2018).
H6 will be investigated by means of mediation analysis using the change scores mentioned in section 3. In the mediation model, BDI change scores will serve as predictor, COM change scores as mediator, and ESC change scores as criterion. Indirect effects will be analyzed with the bias corrected bootstrapping method (1000 bootstrap samples, 95% confidence intervals).
In order to account for possible violations of assumptions, robust procedures (MLR estimates) will be used in all regression analyses.

6) Describe exactly how outliers will be defined and handled, and your precise rule(s) for excluding observations.
The data will be visually inspected for outliers and extreme values. However, only multivariate outliers and extreme values will be excluded. All analyses will be run with and without the identified cases.
As this study intended to explore the association of cognitive motivation and self-regulation with depressive symptoms, only individuals whose BDI scores revealed an at least minimal depressive state (i.e., scores > 8; Hautzinger et al., 2009), will be included in the final analyses.

7) How many observations will be collected or what will determine sample size? No need to justify decision, but be precise about exactly how the number will be determined.
Participants of the study were recruited from in-patients of a psychosomatic rehabilitation center in Germany (approved by German pension insurance institute). Patients, who underwent treatment during the time of data collection were offered to participate in the study. The sample size was thus determined by the time period, in which data collection took place. Since this study was originally set up as a time-limited and exploratory pre-study, which aimed to prepare the ground for the conduction of a large-scale multi-center clinical trial, no explicit sample size was calculated. Sample size depended on the number and willingness of patients, which were hospitalized during the time of study.

8) Anything else you would like to pre-register? (e.g., secondary analyses, variables collected for exploratory purposes, unusual analyses planned?)

see 1): Data have already been collected. However, in order to meet the requirements of hypothesis-driven research, the total sample was split into two subsamples (n1 = n2 = 530), of which only the first subsample has been analyzed on an exploratory base yet. In the course of these analyses, new hypotheses and models were developed. The hypothesis-based analysis of the second subsample is the subject of this pre-registration.
