# Supplementary material for: Cognitive Motivation as a Resource for Affective Adjustment and Mental Health
Source: Front Psychol. 2021 Sep 21;12:581681. doi: 10.3389/fpsyg.2021.581681 (PMC8490806; doi:10.3389/fpsyg.2021.581681)
Supplement: Supplementary file 2 [file Data_Sheet_2.pdf]

Supplementary Material

1 Results of the analyses including all individuals without application of any BDI-II cut off

1.1 Sample 1

Supplementary analyses of sample 1 included n_1 = 495 individuals (M_{age} = 52.20, *SD* = 8.37; 67.27% females) of the original 530 individuals. 35 cases were excluded due to missing values. The median time distance between the first and the second assessment was 33 days (range: 14–77 days).

1.1.1 Cross-sectional analyses

Supplementary Table 1. Descriptive statistics and correlation coefficients between t_1- and t_2-scores of all measures

	COM_t_1	COM_t_2	ESC_t_1	ESC_t_2	CEI_t_1	CEI_t_2	BDI_t_1	BDI_t_2	*Mean*	*SD*	*Range*
COM_t_1	(.90)	**.84**	.54	.50	.91	.77	–.46	–.36	2.80	15.30	-36–36
COM_t_2	**.84**	(.77)	.47	.60	.77	.92	–.44	–.47	3.68	14.81	-35–36
ESC_t_1	.54	.46	(.89)	**.75**	.84	.66	–.55	–.43	0.85	11.55	-34–29
ESC_t_2	.51	.59	**.74**	(.93)	.69	.86	–.54	–.58	3.04	11.35	-33–29
CEI_t_1	.91	.77	.83	.68	(.91)	**.82**	–.57	–.44	3.65	23.65	-54–53
CEI_t_2	.78	.92	.65	.85	**.82**	(.79)	–.54	–.58	6.73	23.45	-66–62
BDI_t_1	–.45	–.44	–.55	–.53	–.56	–.53	(.89)	**.71**	25.43	11.89	0–58
BDI_t_2	–.36	–.47	–.44	–.56	–.44	–.57	**.72**	(.95)	13.04	11.85	0–55

Note. Analyses of the first sample (n_1 = 495). Internal consistencies (Cronbach's α) are presented in the diagonal, Pearson coefficients above, and Spearman's Rank coefficients below the diagonal. Bold-faced coefficients give the retest-reliabilities (interval on average 5 weeks). BDI = depressive symptoms; COM = cognitive motivation; ESC = effortful self-control; CEI = cognitive effort investment; t_1 = before treatment; t_2 = after treatment. For all coefficients $p < .001$ (Bonferroni-adjusted $\alpha = 0.0018$).

1.1.2 Longitudinal analyses

Latent change score modelling

Supplementary Table 2. Results of the latent change score modelling in the first sample

Variable	*b*	SE	95% CI		**β**
			LL	*UL*	
ΔCOM ~ COM_t_1	–0.22***	0.03	–0.28	–0.15	–0.38
ΔCOM ~ ESC_t_1	–0.02	0.05	–0.10	0.07	–0.02
ΔCOM ~ BDI_t_1	–0.09*	0.04	–0.17	–0.01	–0.12
ΔBDI ~ COM_t_1	–0.01	0.03	–0.07	0.04	–0.03
ΔBDI ~ ESC_t_1	–0.05	0.04	–0.14	0.04	–0.06
ΔBDI ~ BDI_t_1	–0.32***	0.04	–0.41	–0.24	–0.43
ΔESC ~ COM_t_1	0.07*	0.03	0.01	0.13	0.14
ΔESC ~ ESC_t_1	–0.40***	0.04	–0.48	–0.33	–0.57
ΔESC ~ BDI_t_1	–0.16***	0.04	–0.23	–0.08	–0.23

Note. n_1 = 495. BDI = depressive symptoms; COM = cognitive motivation; ESC = effortful self-control; t_1 = before treatment; Δ = latent change score (i.e., the change from t_1 to t_2); ~ = predicted by; CI = confidence interval; LL = lower limit; UL = upper limit. * $p < .05$, ** $p < .01$, *** $p < .001$.

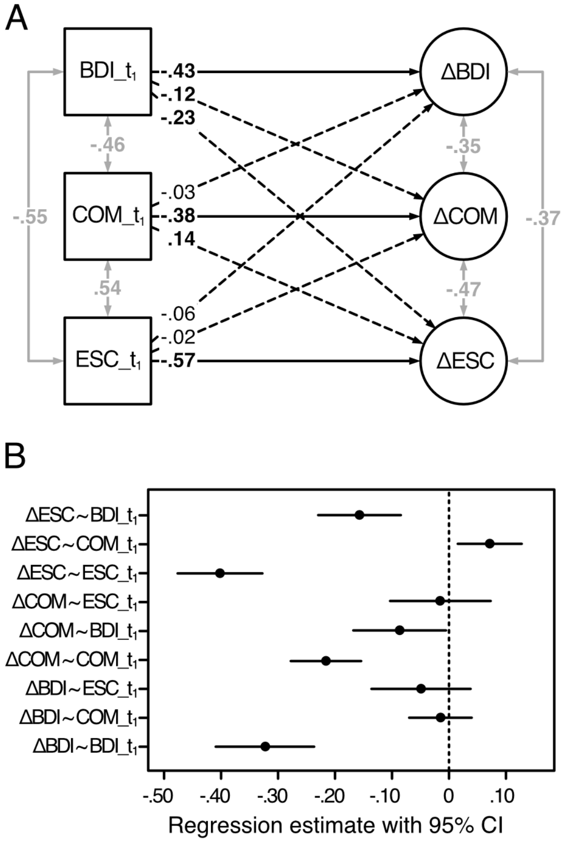

Supplementary Figure 1. Estimated latent change score model of the change scores predicted by the baseline scores. Supplementary analysis based on the first sample (n_1 = 495). BDI = depressive symptoms; COM = cognitive motivation; ESC = effortful self-control; t_1 = before treatment; Δ = latent change score (i.e., the change from t_1 to t_2). Panel A: Standardized regression coefficients are displayed. One-sided solid and dashed arrows refer to the model presented in the main text and indicate directed effects (regression coefficients), two-sided grey arrows indicate undirected relationships (correlations). Coefficients in bold are significant for $p < .05$. Panel B: 95% confidence intervals of the unstandardized regression estimates, based on 1000 bootstrap samples.

Mediation analyses

Supplementary Table 3. Results of the mediation analysis in the first sample

Effect	*b*	SE	95% CI		β
			LL	*UL*	
a	–0.25***	0.03	–0.32	–0.18	–0.07
b	0.72***	0.05	0.63	0.82	0.63
c	–0.06	0.04	–0.13	0.01	–0.01
ab	–0.18***	0.03	–0.23	–0.13	–0.04
total	–0.24***	0.04	–0.32	–0.16	–0.05

Note. n_1 = 495. CI = confidence interval; LL = lower limit; UL = upper limit. a = direct effect of the change in depressive symptoms (ΔBDI) on the change in cognitive motivation (ΔCOM); b = direct effect of ΔCOM on the change in self-regulation (ΔESC); c = direct effect of ΔBDI on ΔESC with mediator (ΔCOM); ab = indirect mediationg effect of ΔBDI on ΔESC via ΔCOM; total = direct effect of ΔBDI on the change in self-regulation without mediation. *** $p < .001$.

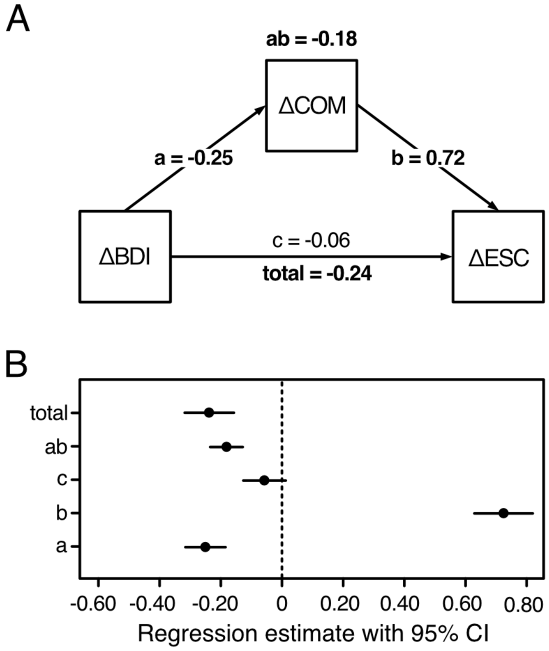

Supplementary Figure 2. Results and model of the supplementary mediation analysis of the first sample (n_1 = 495). BDI = depressive symptoms; COM = cognitive motivation; ESC = effortful self-control; Δ= latent change score (i.e., the change from t_1 to t_2). Panel A: Increasing cognitive motivation completely mediates the association between decreasing depressive symptoms and increasing self-regulation. The following unstandardized effects are displayed: total (direct effect without mediation) = ab (indirect mediating effect) + c (direct effect with mediator). Panel B: 95% confidence intervals of the unstandardized regression estimates, based on 1000 bootstrap samples.

1.2 Sample 2

Supplementary analyses of sample 2 included n_2 = 488 individuals (M_{age} = 51.47, *SD* = 8.88; 67.42% females) of the original 530 individuals. 42 cases had to be excluded due to missing values. The median time distance between the first and the second assessment was 33 days (range: 13–75 days).

Unequal variance t-test revealed no differences between the samples of sample 1 and sample 2 with respect to age ($t_{(975.68)} = 1.32$, $p = 0.186$), gender ($t_{(980.83)} = –0.05$, $p = 0.961$), nor any of the variables' means: COM_t_1 ($t_{(977.88)} = 0.02$, $p = 0.987$), COM_t_2 ($t_{(980.53)} = 0.41$, $p = 0.681$), ESC_t_1 ($t_{(980.04)} = 0.47$, $p = 0.635$), ESC_t_2 ($t_{(977.99)} = 0.50$, $p = 0.617$), BDI_t_1 ($t_{(977.35)} = –0.80$, $p = 0.425$), BDI_t_2 ($t_{(980.79)} = 0.21$, $p = 0.835$).

1.2.1 Cross-sectional analyses

Supplementary Table 4. Descriptive statistics and correlation coefficients between t_1- and t_2-scores of all measures

	COM_t_1	COM_t_2	ESC_t_1	ESC_t_2	CEI_t_1	CEI_t_2	BDI_t_1	BDI_t_2	*Mean*	*SD*	*Range*
COM_t_1	(.88)	**.82**	.58	.54	.91	.77	–.39	–.38	2.79	14.26	-36–35
COM_t_2	**.81**	(.79)	.47	.62	.74	.92	–.37	–.47	3.30	14.28	-36–36
ESC_t_1	.56	.43	(.89)	**.76**	.87	.67	–.48	–.40	0.50	11.75	-32–32
ESC_t_2	.53	.58	**.74**	(.91)	.72	.88	–.47	–.56	2.67	11.83	-33–36
CEI_t_1	.91	.72	.85	.69	(.91)	**.81**	–.49	–.43	3.29	23.17	-68–63
CEI_t_2	.77	.91	.64	.85	**.80**	(.82)	–.47	–.57	5.98	23.49	-69–72
BDI_t_1	–.39	–.36	–.47	–.45	–.48	–.45	(.89)	**.71**	26.01	11.03	1–56
BDI_t_2	–.36	–.44	–.41	–.58	–.43	–.56	**.69**	(.94)	12.89	11.51	0–50

Note. Supplementary analyses of the second sample (n_2 = 488). Internal consistencies (Cronbach's alpha) are presented in the diagonal, Pearson coefficients above, and Spearman's Rank coefficients below the diagonal. Bold-faced coefficients give the retest-reliabilities (interval on average 5 weeks). BDI = depressive symptoms; COM = cognitive motivation; ESC = effortful self-control; CEI = cognitive effort investment; t_1 = before treatment; t_2 = after treatment. For all coefficients $p < .001$ (Bonferroni-adjusted $\alpha = 0.0018$).

1.2.2 Longitudinal analyses

Latent change score modelling

Supplementary Table 5. Results of the latent change score modelling in the second sample

Variable	*b*	SE	95% CI		**β**
			LL	*UL*	
ΔCOM ~ COM_t_1	–0.19***	0.03	–0.25	–0.13	–0.31
ΔCOM ~ ESC_t_1	–0.05	0.04	–0.13	0.03	–0.07
ΔCOM ~ BDI_t_1	–0.10*	0.04	–0.17	–0.02	–0.13
ΔBDI ~ COM_t_1	–0.09**	0.03	–0.15	–0.03	–0.15
ΔBDI ~ ESC_t_1	–0.02	0.04	–0.09	0.05	–0.02
ΔBDI ~ BDI_t_1	–0.32***	0.04	–0.40	–0.23	–0.40
ΔESC ~ COM_t_1	0.11***	0.03	0.05	0.17	0.19
ΔESC ~ ESC_t_1	–0.37***	0.04	–0.44	–0.30	–0.53
ΔESC ~ BDI_t_1	–0.13***	0.03	–0.20	–0.06	–0.18

Note. n_2 = 488. BDI = depressive symptoms; COM = cognitive motivation; ESC = effortful self-control; t_1 = before treatment; Δ= latent change score (i.e., the change from t_1 to t_2); ~ = predicted by; CI = confidence interval; LL = lower limit; UL = upper limit. * $p < .05$, ** $p < .01$, *** $p < .001$.

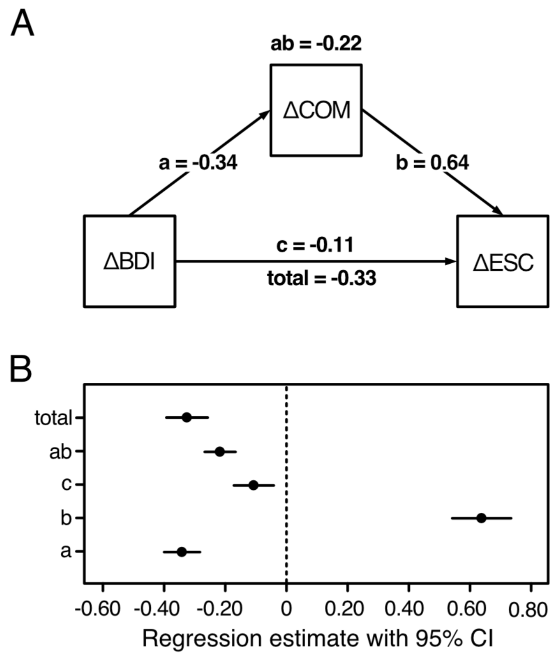

Supplementary Figure 3. Estimated latent change score model of the change scores predicted by the baseline scores. Supplementary analysis based on the second sample (n_2 = 488). BDI = depressive symptoms; COM = cognitive motivation; ESC = effortful self-control; t_1 = before treatment; Δ= latent change score (i.e., the change from t_1 to t_2). Panel A: Standardized regression coefficients are displayed. One-sided dotted and dashed arrows refer to the model presented in the main text and indicate directed effects (regression coefficients), two-sided grey arrows indicate undirected relationships (correlations). Coefficients in bold are significant for $p < .05$. Panel B: 95% confidence intervals of the unstandardized regression estimates, based on 1000 bootstrap samples.

Mediation analyses

Supplementary Table 6. Results of the mediation analysis in the second sample

Effect	*b*	SE	95% CI		**β**
			LL	*UL*	
a	–0.47***	0.04	–0.54	–0.39	–0.10
b	0.37***	0.05	0.27	0.47	0.36
c	–0.34***	0.04	–0.42	–0.26	–0.07
ab	–0.17***	0.03	–0.23	–0.12	–0.04
total	–0.51***	0.04	–0.59	–0.44	–0.11

Note. n_2 = 488. CI = confidence interval; LL = lower limit; UL = upper limit. For all $p < .001$. Effects: a = direct effect of the change in depressive symptoms (ΔBDI) on the change in cognitive motivation (ΔCOM); b = direct effect of ΔCOM on the change in self-regulation (ΔESC); c = direct effect of ΔBDI on ΔESC with mediator (ΔCOM); ab = indirect mediationg effect of ΔBDI on ΔESC via ΔCOM; total = direct effect of ΔBDI on the change in self-regulation without mediation. *** $p < .001$.

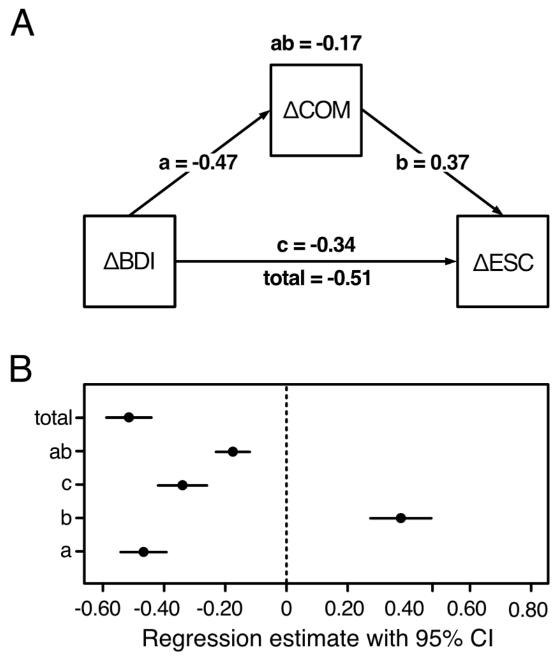

Supplementary Figure 4. Results and model of the supplementary mediation analysis of the second sample (n_2 = 488). BDI = depressive symptoms; COM = cognitive motivation; ESC = effortful self-control; Δ= latent change score (i.e., the change from t_1 to t_2). Panel A: Increasing cognitive motivation partly mediates the association between decreasing depressive symptoms and increasing self-regulation. The following unstandardized effects are displayed: total (direct effect without mediation) = ab (indirect mediating effect) + c (direct effect with mediator). Panel B: *95%* confidence intervals of the unstandardized regression estimates, based on 1000 bootstrap samples.

2 Results of the analyses including only individuals with BDI-II score > 11

2.1 Sample 1

Supplementary analyses of sample 1 included n_1 = 427 individuals (M_{age} = 51.92, *SD* = 8.53; 69.09% females) of the original 530 individuals. 35 cases were excluded due to missing values and 68 cases due to BDI-II scores < 12 (Riedel et al., 2010). The median time distance between the first and the second assessment was 33 days (range: 14–77 days).

2.1.1 Cross-sectional analyses

Supplementary Table 7. Descriptive statistics and correlation coefficients between t_1- and t_2-scores of all measures

	COM_t_1	COM_t_2	ESC_t_1	ESC_t_2	CEI_t_1	CEI_t_2	BDI_t_1	BDI_t_2	*Mean*	*SD*	*Range*
COM_t_1	(.89)	**.82**	.52	.46	.91	.75	–.39	–.30	1.01	14.99	-36– 36
COM_t_2	**.82**	(.75)	.43	.57	.75	.92	–.40	–.44	2.19	14.60	-35–36
ESC_t_1	.51	.43	(.88)	**.72**	.82	.62	–.46	–.37	–0.89	10.99	-34–29
ESC_t_2	.46	.56	**.71**	(.89)	.65	.85	–.48	–.55	1.48	10.85	-33–28
CEI_t_1	.91	.75	.81	.64	(.91)	**.80**	–.48	–.38	0.12	22.71	-54–52
CEI_t_2	.75	.92	.61	.83	**.79**	(.77)	–.48	–.55	3.68	22.61	-66–62
BDI_t_1	–.38	–.40	–.46	–.46	–.47	–.48	(.88)	**.68**	28.33	10.04	12–58
BDI_t_2	–.29	–.45	–.35	–.52	–.36	–.54	**.65**	(.94)	14.69	11.86	0–55

Note. Analyses of the first sample (n_1 = 427). Internal consistencies (Cronbach's alpha) are presented in the diagonal, Pearson coefficients above, and Spearman's Rank coefficients below the diagonal. Bold-faced coefficients give the retest-reliabilities (interval on average 5 weeks). BDI = depressive symptoms; COM = cognitive motivation; ESC = effortful self-control; CEI = cognitive effort investment; t_1 = before treatment; t_2 = after treatment. For all coefficients $p < .001$ (Bonferroni-adjusted $\alpha = 0.0018$).

2.1.2 Longitudinal analyses

Latent change score modelling

Supplementary Table 8. Results of the latent change score modelling in the first sample

Variable	*b*	SE	95% CI		β
			LL	*UL*	
ΔCOM ~ COM_t_1	–0.23***	0.04	–0.30	–0.16	–0.38
ΔCOM ~ ESC_t_1	–0.02	0.05	–0.12	0.07	–0.03
ΔCOM ~ BDI_t_1	–0.13*	0.05	–0.24	–0.03	–0.15
ΔBDI ~ COM_t_1	–0.01	0.03	–0.08	0.05	–0.02
ΔBDI ~ ESC_t_1	–0.07	0.05	–0.17	0.03	–0.08
ΔBDI ~ BDI_t_1	–0.24***	0.05	–0.34	–0.13	–0.27
ΔESC ~ COM_t_1	0.06	0.03	–0.002	0.12	0.11
ΔESC ~ ESC_t_1	–0.41***	0.04	–0.50	–0.33	–0.55
ΔESC ~ BDI_t_1	–0.18***	0.05	–0.27	–0.09	–0.22

Note. n_1 = 427. BDI = depressive symptoms; COM = cognitive motivation; ESC = effortful self-control; t_1 = before treatment; Δ= latent change score (i.e., the change from t_1 to t_2); ~ = predicted by; CI = confidence interval; LL = lower limit; UL = upper limit. * $p < .05$, ** $p < .01$, *** $p < .001$.

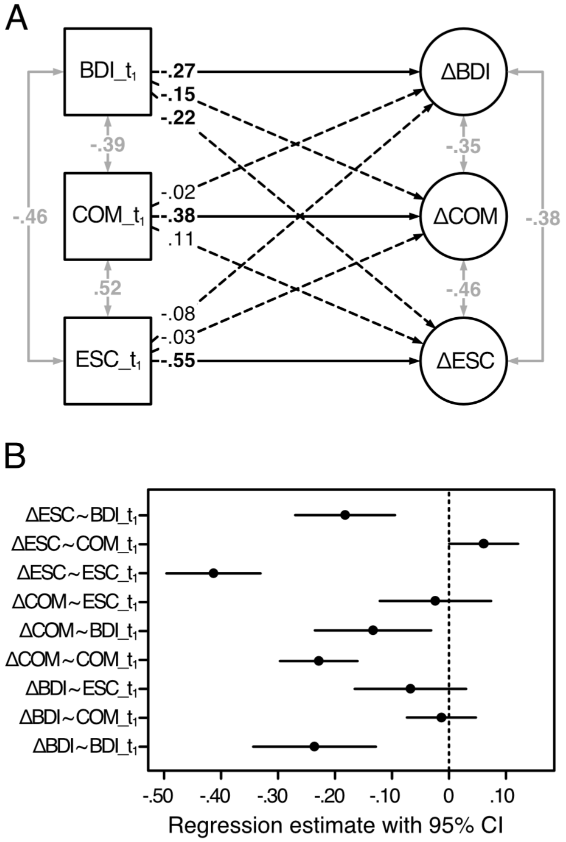

Supplementary Figure 5. Estimated latent change score model of the change scores predicted by the baseline scores. Supplementary analysis based on the first sample (n_1 = 427). BDI = depressive symptoms; COM = cognitive motivation; ESC = effortful self-control; t_1 = before treatment; Δ= latent change score (i.e., the change from t_1 to t_2). Panel A: Standardized regression coefficients are displayed. One-sided dotted and dashed arrows refer to the model presented in the main text and indicate directed effects (regression coefficients), two-sided grey arrows indicate undirected relationships (correlations). Coefficients in bold are significant for $p < .05$. Panel B: 95% confidence intervals of the unstandardized regression estimates, based on 1000 bootstrap samples.

Mediation analyses

Supplementary Table 9. Results of the mediation analysis in the first sample

Effect	*b*	SE	95% CI		**β**
			LL	*UL*	
a	–0.37***	0.03	–0.42	–0.32	–0.09
b	0.50***	0.06	0.39	0.62	0.47
c	–0.18***	0.04	–0.25	–0.11	–0.04
ab	–0.18***	0.03	–0.24	–0.13	–0.04
total	–0.37***	0.03	–0.43	–0.31	–0.08

Note. n_1 = 427. CI = confidence interval; LL = lower limit; UL = upper limit. For all $p < .001$. Effects: a = direct effect of the change in depressive symptoms (ΔBDI) on the change in cognitive motivation (ΔCOM); b = direct effect of ΔCOM on the change in self-regulation (ΔESC); c = direct effect of ΔBDI on ΔESC with mediator (ΔCOM); ab = indirect effect of ΔBDI on ΔESC via ΔCOM; total = direct effect of ΔBDI on the change in self-regulation without mediation. *** $p < .001$.

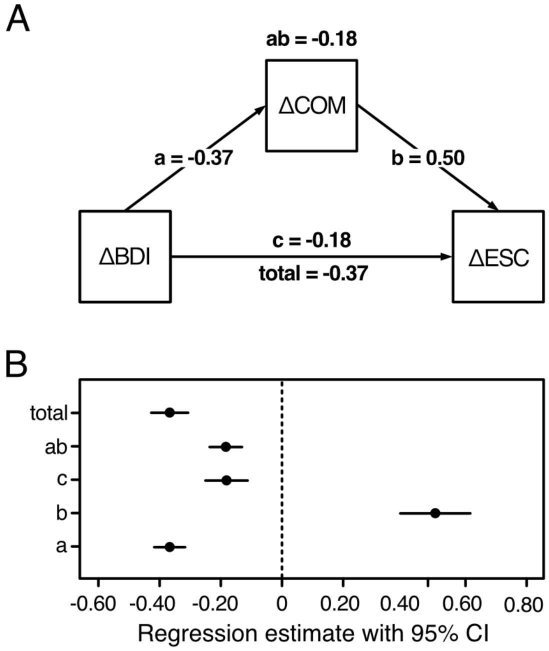

Supplementary Figure 6. Results and model of the supplementary mediation analysis of the first sample (n_1 = 427). BDI = depressive symptoms; COM = cognitive motivation; ESC = effortful self-control; Δ= latent change score (i.e., the change from t_1 to t_2). Panel A: Increasing cognitive motivation partly mediates the association between decreasing depressive symptoms and increasing self-regulation. The following unstandardized effects are displayed: total (direct effect without mediation) = ab (indirect mediating effect) + c (direct effect with mediator). Panel B: *95%* confidence intervals of the unstandardized regression estimates, based on 1000 bootstrap samples.

2.2 Sample 2

Supplementary analyses of sample 2 included n_2 = 443 individuals (M_{age} =51.57, *SD*=8.88; 68.85%females) of the original 530 individuals. 42 cases had to be excluded due to missing values; 45 cases were excluded due to subthreshold BDI scores. The median time distance between the first and the second assessment was 34 days for the Abridged Cognitive Effort Scales and 35 days for Beck Depression Inventory-II (range: 14–75 days).

Unequal variance t-test revealed no differences between the samples of sample 1 and sample 2 with respect to age ($t_{(867.98)}$ = 0.60, p = 0.546), gender ($t_{(866.95)}$ = 0.08, p = 0.940), nor any of the variables' means: COM_t_1 ($t_{(858.40)}$ = –0.57, p = 0.571), COM_t_2 ($t_{(863.60)}$ = –0.08, p = 0.936), ESC_t_1 ($t_{(867.73)}$ = –0.19, p = 0.850), ESC_t_2 ($t_{(867.61)}$ = –0.27, p = 0.790), BDI_t_1 ($t_{(864.81)}$ = 0.70, p = 0.485), BDI_t_2 ($t_{(864.61)}$ = 0.95, p = 0.341).

2.2.1 Cross-sectional analyses

Supplementary Table 10. Descriptive statistics and correlation coefficients between t_1- and t_2-scores of all measures

	COM_t_1	COM_t_2	ESC_t_1	ESC_t_2	CEI_t_1	CEI_t_2	BDI_t_1	BDI_t_2	*Mean*	*SD*	*Range*
COM_t_1	(.88)	**.82**	.55	.52	.91	.76	–.32	–.34	1.57	13.99	-36–35
COM_t_2	**.80**	(.76)	.44	.60	.73	.92	–.32	–.45	2.27	14.11	-36–36
ESC_t_1	.53	.39	(.88)	**.74**	.85	.64	–.39	–.35	–0.74	11.20	-32–30
ESC_t_2	.50	.56	**.72**	(.89)	.70	.87	–.42	–.55	1.68	11.49	-33–36
CEI_t_1	.90	.71	.83	.67	(.91)	**.80**	–.40	–.39	0.82	22.24	-68–59
CEI_t_2	.75	.91	.61	.85	**.79**	(.81)	–.41	–.55	3.96	22.90	-69–72
BDI_t_1	–.31	–.31	–.38	–.40	–.39	–.39	(.88)	**.69**	27.86	9.80	12–56
BDI_t_2	–.31	–.42	–.35	–.55	–.37	–.54	**.65**	(.94)	13.93	11.55	0–50

Note. Analyses of the second sample (n_2 = 443). Internal consistencies (Cronbach's alpha) are presented in the diagonal, Pearson coefficients above, and Spearman's Rank coefficients below the diagonal. Bold-faced coefficients give the retest-reliabilities (interval on average 5 weeks). BDI = depressive symptoms; COM = cognitive motivation; ESC = effortful self-control; CEI = cognitive effort investment; t_1 = before treatment; t_2 = after treatment. For all coefficients $p < .001$ (Bonferroni-adjusted $\alpha = 0.0018$).

2.2.2 Longitudinal analyses

Latent change score modelling

Supplementary Table 11. Results of the latent change score modelling in the second sample

Variable	*b*	SE	95% CI		**β**
			LL	*UL*	
ΔCOM ~ COM_t_1	–0.18***	0.03	–0.24	–0.12	–0.30
ΔCOM ~ ESC_t_1	–0.05	0.04	–0.14	0.04	–0.07
ΔCOM ~ BDI_t_1	–0.11**	0.04	–0.20	–0.03	–0.13
ΔBDI ~ COM_t_1	–0.09**	0.03	–0.16	–0.03	–0.15
ΔBDI ~ ESC_t_1	–0.04	0.04	–0.12	0.04	–0.05
ΔBDI ~ BDI_t_1	–0.25***	0.05	–0.35	–0.16	–0.29
ΔESC ~ COM_t_1	0.11***	0.03	0.05	0.18	0.19
ΔESC ~ ESC_t_1	–0.38***	0.04	–0.45	–0.30	–0.51
ΔESC ~ BDI_t_1	–0.16***	0.04	–0.24	–0.09	–0.19

Note. n_2 = 443. BDI = depressive symptoms; COM = cognitive motivation; ESC = effortful self-control; t_1 = before treatment; Δ= latent change score (i.e., the change from t_1 to t_2); ~ = predicted by; CI = confidence interval; LL = lower limit; UL = upper limit. * $p < .05$, ** $p < .01$, *** $p < .001$.

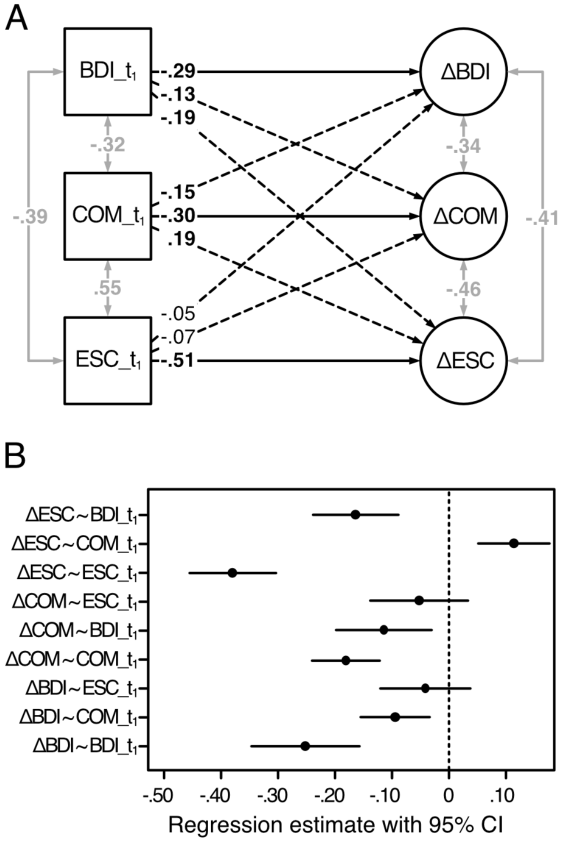

Supplementary Figure 7. Estimated latent change score model of the change scores predicted by the baseline scores. Supplementary analysis based on the second sample (*N* = 443). BDI = depressive symptoms; COM = cognitive motivation; ESC = effortful self-control; t_1 = before treatment; Δ= latent change score (i.e., the change from t_1 to t_2). Panel A: Standardized regression coefficients are displayed. One-sided dotted and dashed arrows refer to the model presented in the main text and indicate directed effects (regression coefficients), two-sided grey arrows indicate undirected relationships (correlations). Coefficients in bold are significant for $p < .05$. Panel B: 95% confidence intervals of the unstandardized regression estimates, based on 1000 bootstrap samples.

Mediation analyses

Supplementary Table 12. Results of the mediation analysis in the second sample

Effect	*b*	SE	95% CI		**β**
			LL	*UL*	
a	–0.46***	0.03	–0.51	–0.40	–0.09
b	0.26***	0.06	0.15	0.37	0.23
c	–0.43***	0.03	–0.50	–0.36	–0.08
ab	–0.12***	0.03	–0.17	–0.06	–0.02
total	–0.55***	0.03	–0.61	–0.49	–0.10

Note. n_2 = 443. CI = confidence interval; LL = lower limit; UL = upper limit. For all $p < .001$. Effects: a = direct effect of the change in depressive symptoms (ΔBDI) on the change in cognitive motivation (ΔCOM); b = direct effect of ΔCOM on the change in self-regulation (ΔESC); c = direct effect of ΔBDI on ΔESC with mediator (ΔCOM); ab = indirect mediationg effect of ΔBDI on ΔESC via ΔCOM; total = direct effect of ΔBDI on the change in self-regulation without mediation. *** $p < .001$.

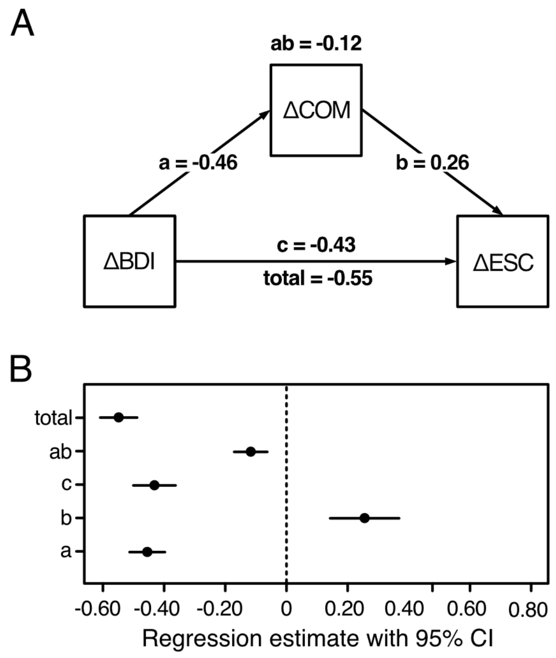

Supplementary Figure 8. Results and model of the supplementary mediation analysis of the second sample (N = 443). BDI = depressive symptoms; COM = cognitive motivation; ESC = effortful self-control; Δ= latent change score (i.e., the change from t_1 to t_2). Panel A: Increasing cognitive motivation partly mediates the association between decreasing depressive symptoms and increasing self-regulation. The following unstandardized effects are displayed: total (direct effect without mediation) = ab (indirect mediating effect) + c (direct effect with mediator). Panel B: *95%* confidence intervals of the unstandardized regression estimates, based on 1000 bootstrap samples.
